# Educational video technology for foot self-care in people with diabetes: a scoping review

**DOI:** 10.1590/1980-220X-REEUSP-2026-0169en

**Published:** 2026-10-30

**Authors:** Amelina de Brito Belchior, Florência Gamileira Nascimento, Victoria Maria Silva Leitão, Thiago Martins de Sousa, Lourival Veras de Oliveira, Cecília Lima de Souza, Sherida Karanini Paz de Oliveira

**Affiliations:** 1 Universidade Estadual do Ceará, Programa de Pós-Graduação em Cuidados Clínicos em Enfermagem e Saúde, Fortaleza, CE, Brazil.; 2 Universidade Estadual do Ceará, Centro de Ciências da Saúde, Departamento de Enfermagem, Fortaleza, CE, Brazil.

**Keywords:** Diabetes Complications, Diabetic Foot, Self Care, Instructional Film and Video, Biomedical Technology

## Abstract

**Objective:**

To map, in the literature, studies that address existing educational videos on foot self-care for people with diabetes.

**Method:**

A scoping review was conducted in accordance with JBI guidelines. The search was performed in nine databases and repositories, with inclusion criteria defined by the PCC (Participants, Concept, and Context) approach. Data collection took place between March and June 2025.

**Results:**

Twenty-four studies distributed across different countries were included, with Brazil and Indonesia predominating (6; 25.0% each). Eight studies (33.3%) described the video name. Regarding the content, specific self-care actions for feet stand out, such as nail care (11; 45.8%). Three studies did not cite the content of the video.

**Conclusion:**

The available studies that address educational videos described in the literature represent a potentially valuable resource for strengthening foot self-care in people with diabetes. They have the potential to improve self-care and expand access to health education. However, its effectiveness depends on increased standardization, professional curation, and validation processes.

## INTRODUCTION

Diabetes Mellitus (DM) is one of the chronic diseases that has long been a major public health problem and is associated with several complications that significantly compromise people’s quality of life. Among these complications, diabetic foot disease stands out, generating significant economic, social, and psychological impacts on healthcare systems, patients, and their families^(1)^. It is estimated that 131 million people worldwide have lower limb complications associated with diabetes, such as peripheral neuropathy and diabetic foot ulcers, representing 1.8% of the global population^(2)^. This constantly growing number, coupled with difficulties in clinical management, limitations in health services, and social inequalities, contributes to amputation rates that are still considered unacceptable^(3)^.

In this context, self-care plays an essential role in the prevention and early identification of foot complications, and structured, organized, and continuous education is fundamental to avoiding ulcers and amputations related to diabetes^(1)^. Preventive measures and educational actions, including through technological interventions, are essential to reduce health risks and enhance feet self-care in people with diabetes mellitus^(4,5)^.

The rapid evolution of technologies renders it essential for the healthcare sector to keep pace with these innovations. However, its effective incorporation into clinical practice requires the development of high-quality materials and the production of systematic evidence on its application^(6)^. Thus, audiovisual materials, due to their accessibility and wide reach, can support the prevention of complications, strengthen patient engagement, and complement traditional health education strategies. By identifying and systematizing these initiatives, the expansion of the use of digital technologies in diabetes care is enhanced, promoting more effective, equitable, and sustainable practices^(7)^.

In this sense, the use of educational videos, as an educational technology in health, is a concrete possibility for support and learning that aims to share messages and guidance in the clearest and most objective way possible, so that the viewer feels motivated and can efficiently reproduce the guidance provided^(8)^. This can benefit the lives of people with diabetes, facilitating learning with correct instructions for feet self-care, improving and transforming the ways they care for themselves, making them more motivated and committed to their health condition^(4)^.

Therefore, it becomes relevant to develop guidance actions on foot self-care for people with diabetes through audiovisual technologies. Thus, the educational videos available in the scientific literature should be found, and their characteristics, potential, content covered, and existing gaps analyzed.

Therefore, this study aims to map, in the literature, the existing educational videos on foot self-care for people with diabetes.

## METHOD

### Design of Study

This is a scoping review conducted according to the recommendations of *Joanna Briggs Institute* (JBI) and structured based on the *checklist Preferred Reporting Items for Systematic reviews and Meta-Analyses extension for Scoping Reviews* (PRISMA-ScR). The research protocol was registered in *Open Science Framework* (OSF) under identification: https://doi.org/10.17605/OSF.IO/BGTHK.

It was developed in nine stages: 1) Identifying the title and research question; 2) Defining eligibility criteria according to the PCC strategy; 3) Selecting evidence sources; 4) Defining the search strategy; 5) Screening and selecting evidence; 6) Extracting data using a specific form; 7) Analyzing and synthesizing the data; 8) Presenting the results; and 9) Summarizing the evidence in relation to the purpose of the review, conclusions, and implications of the findings^(9)^.

### Study Stages

#### First Stage – Identifying the Title and Research Question

In the first stage, the PCC strategy was used^(9)^ considering: Population – people with diabetes; Concept – educational videos; and Context – feet self-care. Thus, the following research question was formulated: “What studies addressing educational videos on foot self-care for people with diabetes are available in the literature?” The secondary question was: What topics are covered in educational videos about foot self-care for people with diabetes?

#### Second Stage – Defining the Eligibility Criteria

The second stage consisted of defining the inclusion and exclusion criteria, according to the PCC strategy. Studies were included that featured videos aimed at people of any age and type of diabetes that addressed foot self-care, distributed in any format, such as online modules, applications, social media, or content stored on mobile devices, in all languages and without time restrictions. Studies involving technologies exclusively for healthcare professionals that did not specify the type of technology used were excluded, as were comments, letters to the editor, abstracts, conference posters, and review studies, due to the limited information available. However, their references were analyzed to identify and select studies that may be relevant for inclusion.

#### Third Stage – Selecting Sources of Evidence

The third stage, selection of evidence sources, was carried out in the following databases and search repositories in the health field: Medical Literature Analysis and Retrieval System Online (MEDLINE) via PubMed, Latin American and Caribbean Literature in Health Sciences (LILACS) via Virtual Health Library (VHL), Web of Science, Scopus, Cumulative Index to Nursing and Allied Health Literature (CINAHL), and Embase. Grey literature was recovered from four sources: Google Scholar, Brazilian Digital Library of Theses and Dissertations (BDTD), Catalog of Theses and Dissertations (CTD) of the Coordination for the Improvement of Higher Education Personnel (CAPES), ProQuest Dissertations and Theses (PQDT).

#### Fourth Stage – Defining the Search Strategy

To define the search strategy, descriptors, keywords, and synonyms were used according to each database, through research in health thesauri and recognized health dictionaries, such as Medical Subject Headings (MESH), Health Sciences Descriptors (DeCS), and EMTREE. It is worth noting that thesis and dissertation filters were applied on the Proquest platform.

To formulate a sensitive search strategy, the ECUS (extraction, conversion, combination, construction, and use) strategy was used^(10)^, along with the Boolean operators OR and AND (Chart 1).

**Chart 1:** Search strategy for constructing the scoping review – Fortaleza, CE, Brazil, 2025^(10)^.

Objective/problem	To map educational videos focused on foot self-care for people with diabetes			
	P	C	C	
Extraction	People with diabetes mellitus	Educational videos	Feet self-care	
Conversion	Diabetes mellitus	educational video	Selfcare	diabetic foot
Combination	Diabetes mellitus, Diabetic Neuropathies, diabetes, diabetic, diabetic patient	Webcasts, video, educational video, infographics, video technology, streaming video, video animation	Selfcare, self-care, self-management;	diabetic foot, Diabetic foot, Diabetic Feet, Ulcer Diabetic foot, diabetic foot wound, diabetic foot ulcer, diabetic foot sore
Construction	(“diabetes mellitus” OR “Diabetic Neuropathies” OR diabetes OR diabetic OR “diabetic patient”)	(Webcasts, OR vídeo OR “educational video” OR infographics OR “video technology” OR “streaming video” OR “video animation”)	(Selfcare OR self-care OR self-management OR selfmanagement) AND (“diabetic foot” OR Diabetic foot” OR “Diabetic Feet” OR “Ulcer Diabetic foot” OR “diabetic foot wound” OR “diabetic foot ulcer” OR” diabetic foot sore”)	
#Use 1	(“diabetes mellitus” OR “Diabetic Neuropathies” OR diabetes OR diabetic OR “diabetic patient”) AND (Webcasts, OR vídeo OR “educational video” OR infographics OR “vídeo technology” OR “streaming video” OR “video animation”) AND (Selfcare OR self-care OR self-management OR selfmanagement) AND (“diabetic foot” OR Diabetic foot” OR “Diabetic Feet” OR “Ulcer Diabetic foot” OR “diabetic foot wound” OR “diabetic foot ulcer” OR “diabetic foot sore”).			
#Use 2	(“diabetes mellitus OR “neuropatias diabéticas” OR diabetes OR diabético) AND (Webcast OR vídeos OR “vídeos educacionais” OR Infograficos OR ”animação em vídeo” OR “tecnologia em vídeo”) AND (autocuidado OR autogerenciamento) AND (“pé diabético” OR “pés diabéticos” OR “úlcera do pé diabético”).			

#### Fifth Stage – Screening and Selecting Evidence

The selection of studies was carried out using Rayyan software which allows for blind and independent screening among reviewers, proceeding with the analysis of titles, abstracts, and full reading of studies found in the literature, removing duplicates.

#### Sixth Stage – Extracting Data Using a Specific Form

In the sixth stage, data extraction using a specific form, two examiners independently participated in the eligibility assessment of the studies using the software Endnote web (https://www.myendnoteweb.com/EndNoteWeb.html) and Excel spreadsheets for managing studies. Initially, the examiners conducted a screening based on reading the titles and abstracts. Following a consensus meeting, the selection of articles was confirmed, with exclusions justified according to the established criteria. The studies were read in full, and then the references were analyzed for the inclusion of new studies. This stage took place between March and June 2025.

Data extraction and management were carried out through mapping, containing characterization information such as authorship, year, country, objective title, type of study, language, level of evidence based on the Melnyk and Fineout-Overholt hierarchical classification^(11)^, and video features.

#### Seventh Stage – Analyzing and Synthesizing Data

In the seventh stage, data analysis and synthesis, agreement was reached among the reviewers through analysis of the Cohen’s Kappa coefficient, adopting the following parameters: 0–0.20, none; 0.21–0.39, minimal; 0.40–0.59, weak; 0.60–0.79, moderate; 0.80–0.90, strong; above 90, almost perfect^(12)^.

During the data extraction process, a critical summary was created, synthesizing all this information. It is also important to emphasize that the level of evidence according to this reference is determined by the methodological characteristics of the studies; the higher the level of evidence, the better the representation of studies with a higher degree of reliability for decision-making. Furthermore, characteristics of the textual production of the studies themselves were also considered, such as the name of the video created, characterization of the technology, duration, target audience, and language used.

The results were presented using graphic materials such as charts, tables, and figures, and analyzed in light of relevant current literature.

The eighth stage, presentation of results, and the ninth, summary of the evidence in relation to the purpose of the review, conclusions and implications of the findings, are arranged in sequence.

### Ethical Aspects

In conducting this study, all ethical principles were respected. All authors of the analyzed articles were properly referenced, in accordance with Copyright Law No. 9.610, of February 19, 1998^(13)^. Data and information from the research were presented accurately.

During the preparation of this article, the authors did not use text generation tools and/or Artificial Intelligence (AI).

## RESULTS

The search identified 1,906 potentially relevant results, of which 653 were removed due to duplication. The remaining 1,253 studies were selected based on title and abstract. Of this total, 50 studies were included for full-text analysis by two independent reviewers. Following full reading, 31 studies were excluded for not answering the research question, and 19 studies remained.

Based on a search of the reference lists, five studies were identified to form the sample, resulting in 24 studies included in this scoping review. Figure 1 illustrates the selection process.

**Figure 1:**
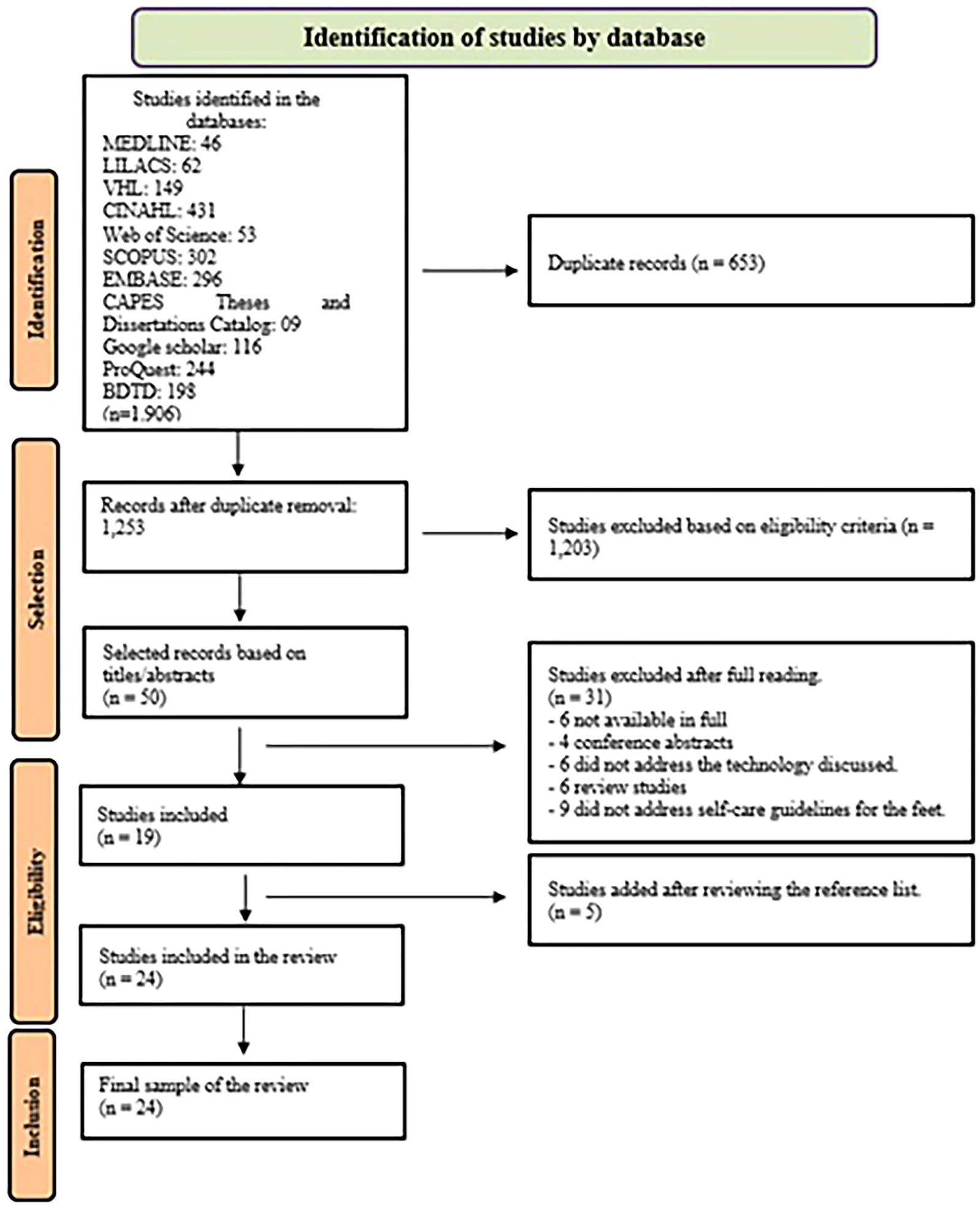
PRISMA-ScR flowchart for selecting scoping review studies – Fortaleza, CE, Brazil, 2025.

**Source:** Research data (2025), adapted from Page et al.^(14)^.

The included studies are distributed across different countries, with predominance of Brazil^(4,15–19)^ and Indonesia^(20–25)^ (6;25.0% each), followed by the United States^(26–28)^, (3;12.5%), Türkiye^(29,30)^ (2;8.3%), Portugal^(29)^, Tunisia^(30)^, India^(31)^, Morocco^(32)^, Netherlands^(33)^ (1;4.1% each). Two studies were conducted in two countries: Saudi Arabia and the United Kingdom^(34)^ and Saudi Arabia and Canada^(35)^ (1;4.2% each).

The earliest study was published in 1987^(33)^, (1;4,1%), with a higher prevalence in the years 2022^(17–19,34,36)^ and 2023^(15,16,20,29,30)^ (5;20.8 each). Others had a lower frequency of publication in 2011^(27,28)^, 2019^(24,26)^ (2; 8.3% each), 1987^(33)^, 2014^(25)^, 2015^(35)^, 2017^(32)^, 2021^(21)^, 2024^(4)^ (1;4.1% each).

The most frequent type of publication was articles^(4,17,19–37)^ (21;87.5%) followed by dissertation^(15,16,18)^ (3;12.5%), published in English^(24,26–29,31–37)^ (12;50.0%), Portuguese^(4,15–19)^ (6;25.0%), Indonesian^(20–23,25)^ (5;20.8%), and French^(30)^ (1;4.1%).

Regarding the method, quasi-experimental studies stand out^(20–25,28,30)^(8;33.3%), followed by methodological^(15–19)^ and Randomized Clinical Trial^(27,29,31,32,37)^ (5;20.8% each). Four articles^(26,34–36)^ (16.6%) rated videos about foot self-care as a source of information available on the platform YouTube and one article^(29)^ (4.1%) refers to a Randomized Clinical Trial protocol.

The studies found most frequently presented evidence level II^(27,29,31,32,37)^ (5;20.8%), III^(20–26,28,30)^ (9;37.5%), and VI^(4,15–19,33–36)^ (10;41.6%).

Chart 2 presents the characterization of the studies included in the review, regarding authorship, year of publication, country, title, objectives, sample, language, method, and level of evidence.

**Chart 2:** Characterization of the studies selected for the scoping review – Fortaleza, CE, Brazil, 2025.

ID	Author (year)/country	Title	Objective	Sample	Language	Method/level of evidence
E1	Andrade et al. (2024)^(4)^. Brazil	Percepção dos usuários com Diabetes sobre o Autocuidado com os Pés: Uma análise qualitativa	To evaluate the perception of the use of educational videos for foot self-care among patients with type 2 diabetes in primary care in the state of Amazonas.	20 people with T2DM.	Portuguese	Qualitative study (Article)/VI
E2	Ferreira et al. (2023)^(29)^. Portugal	Educational Interventions on Diabetic Foot Self-Care: A Study Protocol for a Pragmatic Randomized Controlled Trial	To analyze the impact of different educational strategies – an instructional video.	54 people with DM and foot ulcers, 18 in each group.	English	Randomized clinical trial (Article)/II
E3	Octaviana and Hati (2023)^(20)^. Indonesia	Analisis Pengaruh Edukasi Melalui Media Video terhadap Tingkat Pengetahuan Pasien Diabetes Melitus Tentang Perawatan Kaki di Puskesmas	To analyze the effectiveness of foot care education through videos on the knowledge level of patients with diabetes mellitus.	60 people with DM	Indonesian	Quasi-experimental study (Article)/III
E4	Oliveira (2023)^(15)^. Brazil	Construção e validação de vídeo educativo para prevenção do pé diabético centrado no apoio social	To create and validate an educational video for the prevention of diabetic foot, focusing on the social support of people with DM.	26 specialists (healthcare professionals)	Portuguese	Methodological study (Dissertation)/VI
E5	Yazidi et al. (2023)^(30)^. Tunisia	Place des supports vidéo disponibles en ligne dans l’éducation thérapeutique des diabétiques: étude évaluative chez une population de diabétiques à haut risque podologique	To evaluate the impact of an online educational video on improving the knowledge of diabetic patients at high risk of foot problems regarding the prevention of foot injuries.	40 people with diabetes at high risk in their feet.	French	Quasi-experimental study (Article)/III
E6	Siqueira (2023)^(16)^. Brazil	Vídeo Educativo: atividades de autocuidado para prevenção de complicações nos pés de pessoas idosas com diabetes	To create an educational video about self-care activities for preventing foot complications in older people with diabetes.	9 specialists (nurses)	Portuguese	Methodological study (Dissertation)/VI
E7	Almaqhawi et al. (2023)^(34)^. Saudi Arabia and the United Kingdom	Evaluation of quality of diabetic foot examination on YouTube	To evaluate the educational quality and reliability of publicly available YouTube videos about foot examinations in people with diabetes, to verify whether or not YouTube is a reliable source of health information for patients.	100 YouTube videos	English	Descriptive study (Article)/VI
E8	Doğan and Pehlivan (2022)^(36)^. Türkiye	Diabetic foot care training and the presence of nurses in Turkish YouTube videos	To analyze the content of Turkish YouTube videos as a source of information on training in foot care for diabetic patients and the presence of nurses in these videos.	87 YouTube videos	English	Descriptive study (Article)/VI
E9	Menezes et al. (2022)^(17)^. Brazil	Produção e validação do curta-metragem Pés que te quero®: tecnologia educacional para pessoas com diabetes	To describe the production and validation of short film-type educational technology for the prevention of diabetic foot ulcers.	31 nursing specialists, 6 communication specialists, and 15 people with type 2 diabetes.	Portuguese	Methodological study (Article)/VI
E10	Nascimento (2022)^(18)^. Brazil	Construção e validação do vídeo cordel “ô pé arretado” como tecnologia educativa para o pé diabético	To construct and validate a cordel literature (a type of Brazilian folk literature) with video presentation as an educational technology for the prevention of diabetic foot.	8 nursing students and 6 specialists for content and appearance validation, and four people with DM to evaluate usability.	Portuguese	Methodological study (Dissertation)/VI
E11	Meneses et al. (2022)^(19)^. Brazil	Desenvolvimento e validação de vídeo educativo sobre cuidados podiátricos para prevenção de úlceras em idosos com diabetes	To develop and validate an educational video about podiatric care for the prevention of diabetic foot in older people.	10 experts.	Portuguese	Methodological study (Article)/VI
E12	Wicahyani et al. (2021)^(21)^. Indonesia	Aplikasi Edukasi Berbasis Video Untuk Meningkatkan Dukungan Keluarga Mencegah Komplikasi Kaki Diabetes	To investigate the effectiveness of video-based education in increasing family support for the prevention of foot complications in patients with type 2 diabetes mellitus.	80 people with diabetes and their families.	Indonesian	Quasi-experimental study (Article)/III
E13	Hamzah et al. (2020)^(22)^. Indonesia	The effectiveness of foot care education video in Makassar language to increase patients’ knowledge related to foot care in community	To determine the effectiveness of an educational video on foot care, in the Makassar language, in improving the knowledge of patients with DM on the subject.	30 people with DM without foot ulcers	Indonesian	Quasi-experimental study (Article)/III
E14	Dinçer and Bahçecik (2020)^(37)^. Türkiye	The effect of a mobile application on the foot care of individuals with type 2 diabetes: A randomised controlled study	To develop a mobile educational application on foot care for diabetics (M-DFCE) with animation support for use by individuals with type 2 diabetes and to identify its effects on knowledge, self-efficacy, and foot care behavior.	130 people with diabetes (65 in the experimental group and 65 in the control group).	English	Randomized clinical trial (Article)/II
E15	Nurjanna et al. (2020)^(23)^. Indonesia	Perbandingan pengetahuan self efficacy perawatan kaki pada pasien Diabetes Melitus tipe II setelah menggunakan video edukasi di puskesmas paccerakkang kota makassar	To compare the knowledge about self-confidence in foot care in patients with type 2 Diabetes Mellitus after the use of educational videos at the Paccerakkang Community Health Center in Makassar City.	20 patients with DM	Indonesian	Quasi-experimental study (Article)/III
E16	Abrar et al. (2020)^(24)^. Indonesia	Development and evaluation educational videos of diabetic foot care in traditional languages to enhance knowledge of patients diagnosed with diabetes and risk for diabetic foot ulcers	To develop an educational video about diabetic foot care in traditional languages (Bukinese and Macassarese) and evaluate the effect of these videos on patient knowledge.	7 specialist nurses for video content development, three for content validity, and 20 people from each ethnic group with diabetes mellitus at risk of foot ulcers.	English	Quasi-experimental study (Article)/III
E17	Smith et al. (2019)^(26)^. EUA	Analysis of YouTube as a Source of Information for Diabetic Foot Care	To alert physicians about the quality of information on diabetic foot care on YouTube and to establish whether YouTube is a useful source of information for patients with diabetic foot problems.	87 YouTube videos	English	Descriptive study (Article)/VI
E18	Rahaman et al. (2018)^(31)^. Índia	Effectiveness of a Patient Education Module on Diabetic Foot Care in Outpatient Setting: An Open-label Randomized Controlled Study	To develop an educational module for patients on diabetic foot care and assess their effectiveness in knowledge and in the practice of foot care for patients with diabetes.	127 (participated in all stages: 101 patients with type 1 and type 2 diabetes) IG: 63 (51 participated in all stages). CG: 63 (51 participated in all stages).	English	Randomized clinical trial (Article)/II
E19	Adarmouch et al. (2017)^(32)^. Morocco	Short-term effectiveness of a culturally tailored educational intervention on foot self-care among type 2 diabetes patients in Morocco	To evaluate the effectiveness of a culturally adapted self-care education intervention for foot care self-management practices in patients with type 2 diabetes and to identify factors associated with variations in these practices.	199 people with DM were recruited and participated in the first assessment, and 133 completed the second assessment.	English	Randomized clinical trial (Article)/II
E20	Abedin et al. (2015)^(35)^. Saudi Arabia and Canada	YouTube as a source of useful information on diabetes foot care on diabetes foot care	To analyze the use of YouTube as an effective source of information on diabetic foot care.	89 YouTube videos	English	Documentary research (Article)/VI
E21	Setyorini et al. (2014)^(25)^. Indonesia	Edukasi perawatan kaki melalui media guidance motion picture dan leaflet terhadap perilaku perawatan kaki non ulkus pada penderita Diabetes Melitus Tipe 2 di Puskesmas Loceret	To evaluate the effectiveness of foot care education using audiovisual resources and leaflets on ulcer-free foot care for patients with T2DM at the Loceret Community Health Center.	30 patients with T2DM	Indonesian	Quasi-experimental study (Article)/III
E22	Grady et al. (2011)^(27)^ USA	Using message framing to achieve long-term behavioral changes in persons with diabetes	To examine the impact of how information is presented in an educational program on proper foot care and its importance in preventing diabetes complications, considering long-term changes in knowledge, attitudes, and behavior regarding foot care.	155 people with diabetes for 5 years or more and no history of foot problems.	English	Randomized clinical trial (Article)/II
E23	Gravely et al. (2011)^(28)^. USA	Comparison of three types of diabetic foot ulcer education plans to determine patient recall of education	To compare current written educational material, used with an educational video and a combination of written and video material to determine the patient’s recall of the education.	30 people with DM.	English	Quasi-experimental study (Article)/III
E24	Bakker (1987)^(33)^. Holanda	Video Education on Foot-Care	To educate patients and staff and to verify whether a hospital, with the assistance of a professional video director, would be able to produce a quality video on a reduced budget.	40 patients with DM	English	Descriptive observational study (Article)/VI

Legend: DM: diabetes mellitus; T2DM: type 2 diabetes mellitus; T1DM: type 1 diabetes mellitus; CG: control group; IG: intervention group; M-DFCE: Mobile Diabetic Foot Care Education.

Source: Authors (2025).

It was observed that in eight studies (33.3%) the names of the videos were described: “Verdadeiro ou Falso (True or False)? and “Diabetes e Cuidados diários com os pés (Diabetes and Daily Foot Care)”^(4)^, “Pés de quem Cuido (The Feet of Those I Care For)”^(15)^, “Autocuidado com os pés de idosos com diabetes (Self-care for the feet of older people with diabetes)”^(16)^, “Pés que te quero (The feet I love)”®^(17)^, “Ô pé arretado (Oh, what an awsome foot)”^(18)^, “No rastro da prevenção: Uma narrativa sobre o diabetes e os cuidados com os pés na pessoa idosa (In the wake of prevention: A narrative about diabetes and foot care in the elderly)^(19)^, “*Guidance Motion Picture* (GMP)”^(25)^ and the *Mobile Diabetic Foot Care Education* (M-DFCE)^(37)^, which contains animated videos about self-care guidelines for the feet.

Among the videos whose time was reported^(15–19,21,22,24,26,27,29–31,33–37)^ (18;75.0%), the duration ranged from 2 minutes and 51 seconds^(18)^ up to 28^(33)^ and 30 minutes^(35)^. Videos lasting between 3 and 10 minutes predominated^(16,18,19,22,24,26,27,29–31,37)^ (11;45.8%). It is noteworthy that of the four studies (16.6%) that evaluated the videos of YouTube^(23,31,34,36)^, two (8.3%) brought the average video length, 6.8 ± 6.6’^(34)^ and 8.08 ± 13.27’^(36)^ and two (8.3%), the maximum video length available on the platform, up to 10’^(26)^ and up to 30’^(35)^.

Regarding the intervention performed in the studies, the pre- and post-test design was prevalent^(20–24,28,30,32,33)^ (9;37.5%) with the application of questionnaires for evaluation before and after exposure to the educational video. Other studies have compared the use of videos with printed materials, traditional guidance, or standard treatment^(25,29,31,37)^ (4;16.6%), while some focused on the validation of the content and appearance of the videos by experts^(15,16,19)^ (3;12.5%), and by experts and target audience^(17,18)^ (2;8.3%). Studies analyzing the quality and usefulness of videos available online were also identified^(26,34–36)^ (4;16.6%). One study^(4)^ (4.1%) applied a questionnaire after the patient watched two educational videos, with the aim of understanding users’ perception of foot care before and after watching the videos. Another study^(27)^ (4.1%) showed two videos to different groups, one with prevention messages focusing on gains, the other on losses, to promote behaviors that maintain health in people with diabetes. An article^(29)^ (4.1%) brought a study protocol on the impact of two different educational strategies, without information on the results.

The main results reported include increased knowledge about foot care^(4,20,22–25,28,30,31,33,37)^ (11;45.8%), improvement in self-care practices^(4,20,32,33,37)^ (5;20.8%), and positive attitude changes^(4,21)^ (2;8.3%).

One study^(21)^ (4.1%) investigated the effectiveness of a video for preventing foot complications in patients with T2DM in increasing family support and revealed that educational videos on examining the feet of patients with diabetes can modify attitudes and increase family support in carrying out foot care for these patients.

Regarding studies that evaluated online videos, the results describe the proportion of videos considered useful, moderately useful, or misleading, as well as the characteristics of the content-producing sources^(26,34–36)^ (4;16.6%).

Chart 3 presents a summary of the main topics covered, interventions, and main results of the studies included in the review.

**Chart 3:** Main topics covered, intervention, and study results – Fortaleza, CE, Brazil, 2025.

ID	Name	Time	Intervention	Key results
E1^(4)^	Verdadeiro ou Falso? and Diabetes e Cuidados diários com os pés	Not described	The participants were visited in their homes by a researcher. During the interviews, participants were in close proximity to a relative or acquaintance. The questions were structured to understand users’ perceptions of foot care before and after watching the videos.	Three categories and two thematic subcategories emerged: 1) ‘prior knowledge’ with the subcategories ‘fragility in professional guidance’ and ‘videos as tools for remembering’; 2) ‘assimilation of language and audiovisual format’; 3) ‘possibilities of impact on self-care’
E2^(29)^	Not described	~ 6’	Instructional video (IG 1) compared with an informational leaflet on foot care with real-time guided reading (IG 2) and standard care (CG) – on adherence to and knowledge of foot care in people with diabetes and on patients’ perception of their foot health over time.	Not described
E3^(20)^	Not described	Not described	A pre-test and a post-test were applied. In the CG, no educational videos were shown, and a questionnaire about foot care was administered in weeks 1 and 4. In the IG group, the questionnaire was administered starting in week 1, and in weeks 2 and 3, an educational video about foot care was shown. In week 4, the questionnaire was administered again.	The Mann-Whitney test results before viewing the video were 0.957, with no significant difference between the IG and CG. After the exhibition, the result was 0.000, indicating a significant difference between the IG and CG. The use of educational videos in education has significantly increased knowledge and self-care of the feet in patients with T2DM.
E4^(15)^	“Pés de quem cuido”	17’	It was not carried out.	In the analysis of the CVI of all subcategories, they were considered valid and reliable. The third version of the script received a CVI score of 0.94. Among the main considerations made by the judges in the validation process, the appropriateness of the characters’ language and the reduction of the texts (characters’ dialogue) stand out.
E5^(30)^	Not described	3’31”	A questionnaire was administered to assess theoretical and practical knowledge about preventing foot injuries in people with diabetes mellitus. Next, the patients watched the video. A reassessment of knowledge, using the same questionnaire, was carried out on the same day.	Over 90% of people stated they were “satisfied” or “very satisfied” with the educational video. Significant improvement was observed in knowledge about preventing foot injuries in people with diabetes mellitus. The impact of the video on reducing the frequency of chronic ulcers and amputations still needs to be determined.
E6^(16)^	Autocuidado com os pés de idosos com diabetes	6’5”	It was not carried out.	First, 16 articles that provided evidence for the prevention of foot complications in older people with diabetes were assessed; this information was then used as a framework for the production of the educational video. After evaluation by nine experts, an overall CVI score of 0.90 was obtained.
E7^(34)^	Not applicable	Average of 6.8 ± 6.6	Not applicable	The most frequent content category was foot examination (58 videos), followed by specific information about the disease (53 videos). The most common source of upload was doctors (28%) and the least common was patients (1%).
E8^(36)^	Not described	Average of 8.08 ± 13.27	Not applicable	Of the videos evaluated, 8% were rated as very useful, 33.3% as moderately useful, 51.7% as somewhat useful, and 6.9% as not useful. It is noteworthy that 4.6% contained misleading information. The instructor was a nurse in only 12.6% of these videos.
E9^(17)^	“Pés que te quero”	24’7”	It was not carried out.	The video script had a total CVI of 0.95, with an internal reliability of 0.849 as verified by Cronbach’s alpha. Communication experts validated it with a CVI of 0.97, and the target audience scored 0.95 (clarity) and 0.97 (relevance).
E10^(18)^	“Ô Pé arretado”	2’51”	It was not carried out.	In the validation with technical judges, there was 100% agreement on the simplicity, clarity, and relevance of the content, and a CVCt of 1.0 for the cordel video script. In addition to 100% agreement from clinical practice judges regarding the simplicity, clarity, and relevance of the content and appearance of the video-cordel, they also agreed that the educational material was suitable for self-care in the prevention of diabetic foot, as well as complications of the disease.
E11^(19)^	“No rastro da prevenção: Uma narrativa sobre o diabetes e os cuidados com os pés na pessoa idosa”	4’33”	It was not carried out.	There was unanimous agreement among the experts for most of the criteria. Nevertheless, according to the criterion adopted for validating the video content, which was the overall CVI, calculated by averaging the CVIs of each item, no changes to the proposed video content were deemed necessary, considering the video content valid with 99.38% agreement among the judges (CVI = 0.99).
E12^(21)^	Not described	12’	A pre-test was conducted via WhatsApp, followed by the sending of the educational video. After watching the video, participants independently performed the foot examination and submitted a video recording of their examination. The post-test was conducted online one week after the pre-test, using a form from Google.	Educational videos about the screening of the feet of patients with diabetes can change attitudes and increase family support in providing foot care for these patients. Based on the results of the Wilcoxon statistical analysis, the p-value was 0.009 (p < 0.05), with a median score of total family support of 77 before and 81.6 after the intervention.
E13^(22)^	Not described	5’15”	A pre-test and post-test were applied to a single group before and after viewing the video of the research by Abrar et al. (2020).	The results revealed an increase in good knowledge (36.7%–90%) and a decrease in moderate knowledge (56.7%–10%) and insufficient knowledge (6.7% to 0%).
E14^(37)^	*Mobile Diabetic Foot Care Education* (M-DFCE)	6 sections, each lasting approximately 1’3”	IG: received guidance on foot care for diabetic patients through a mobile application. CG: received guidance on diabetic foot care in accordance with the hospital’s clinical guidelines. The video was shown once at the beginning of the Health Education session, and a follow-up was conducted one month later.	There was a significant increase in the level of knowledge of IG patients about foot care (p = 0.001). There was no significant increase in the CG knowledge score between the first and last assessments (p = 0.905). There was no difference between the groups in the initial assessment; however, participants in the IG who received M-DFCE with animation support showed significantly higher levels of knowledge, self-efficacy, and foot care behavior compared to the CG.
E15^(23)^	No information	Not described	Application of educational video on foot care (pre- and post-test)	The sample’s knowledge about foot care before the educational video averaged 6.40 ± 1.789, median of 7.0, and the knowledge score after the video reached an average of 10.70 ± 0.571, median of 11.00 (p = 0.000).
E16^(24)^	Not described	>6’	The pre-test was distributed to participants before the video was shown. The video was played back using an image projector for less than 6 minutes. The post-test was conducted using the same questionnaires.	Participants’ knowledge of foot care increased significantly after watching educational videos in both languages, Makassarese (p = 0.001) and Bugisese (p = 0.001).
E17^(26)^	Not described	>10’	Not applicable	Of the 87 videos included, 56 (64.4%) were rated as useful and 31 (35.6%) as not useful/misleading. A significant difference was observed in the average duration of useful videos compared to non-useful/misleading videos (3.33 versus 1.73 min; p < 0.0001). There was no significant difference in terms of popularity metrics (likes, views, subscriptions, etc.) between the helpful and non-helpful/misleading videos.
E18^(31)^	No information	9’	The IG received an audiovisual video as part of the educational module on foot care. CG received routine care.	The knowledge scores in the IG at the first, second, and third consultations were 9.8 ± 1.8, 10.2 ± 1.6, and 11.0 ± 1.7, respectively. The knowledge scores in the CG at the first, second, and third consultations were 9.9 ± 1.7, 9.8 ± 1.6 e 10.0 ± 1.8, respectively. The change in knowledge score was statistically significant (p < 0.001) at the third visit compared to the first in the intervention group, but not in the CG (p = 0.62). The practice score also improved significantly (p < 0.001) in the IG at the second consultation, but not in the CG.
E19^(32)^	Not described	No information	The intervention consisted of an interactive group discussion using different materials: a narrative video, a PowerPoint presentation, and a printed guide. Pre- and post-testing were conducted.	The mean foot care score increased from 3.5 ± 2.9 days to 5.9 ± 1.8 days one month after the intervention (mean range 2.4 ± 3.1 days; p < 0.001). A favorable variation was found in 75 (37.7%) participants.
E20^(35)^	Not described	Up to 30’	Not applicable	The two most frequently mentioned foot care practices in the videos were “checking feet daily” (n = 34; 38.20%) and “wearing appropriate shoes and socks” (n = 26; 29.21%). Only 14.6% of the videos contained useful information, excluding partially useful information. These 14.6% were provided by podiatrists or clinics specializing in foot care. Only 10.1% of the total videos were from general users.
E21^(25)^	“Guidance Motion Picture” (GMP)	Not described	The IG (film orientation + information leaflet), CG positive 1 (film orientation) and CG positive 2 (information leaflet).	Knowledge was significant for the IG (p = 0.005), for the positive CG 1 (p = 0.011) and for the positive GC 2 (p = 0.097). The attitude analysis was significant for all groups (p = 0.005). The analysis of the action was significant for the IG (p = 0.005) and for the control group positive 1 (p = 0.005), the positive control group 2 (p = 0.011). There are differences in effectiveness between the combination of educational video + informational leaflet and the educational video alone, in relation to increasing knowledge (p = 0.047), attitude (0.022), and action (p = 0.037).
E22^(27)^	Not described	~10’	The participants were randomly divided into two groups of equal size: G1 watched the version of the educational video that focused on the benefits. G2 watched the version focusing on the losses. Each participant completed 4 test sessions: before watching the video (demographic data, attitude, knowledge, and behavior), immediately after watching the video (attitude and knowledge), and at 3- and 6-month follow-ups (attitude, knowledge, and behavior).	Videos with preventative messages that focus on benefits are more effective than messages that focus on losses in promoting healthy behaviors in people with diabetes.
E23^(28)^	Not described	Not described	The participants were divided into 3 groups. The CG received the written educational material currently provided by the hospital to patients with diabetes. The IG received video training that covered the same content as the written material. A third group received education through written guidance and video. The video was watched 1 to 3 times while the person was in hospital.	Most patients showed significant change in post-educational tests using the video education tool (IG). A follow-up study is required to provide more evidence of patient preferences regarding education (video only or video + written leaflet).
E24^(33)^	Not described	28	To test the usefulness of the program, a questionnaire was developed. Patients were asked to answer the same eight questions (yes/no) before and after watching the video.	After viewing the video, the number of people who knew the answers to the questions about foot care increased from 69% to 91%. After 6 months, most participants emphasized that they had followed at least some of the self-care recommendations for their feet, which they had not done previously.

Legend: DM: diabetes mellitus; T2DM: type 2 diabetes mellitus; T1DM: type 1 diabetes mellitus; CG: control group; IG: intervention group; M-DFCE: Mobile Diabetic Foot Care Education.

Source: Authors (2025).

Regarding the content covered in the videos, the studies presented general guidelines and guidelines on diabetes^(4,15,16,18,19,25,26,30,31,34–36)^ (12;50.0%), self-care of the feet, in general, without specifying guidelines^(4,16–18,20,23,25–27,29–31,37)^ (13;54,1%). Regarding the self-care actions described in the video, nail care predominated^(15–17,19,22,24,26,30,32,35,36)^ (11;45.8%) followed by foot inspection^(15–17,22,24,26,30,34–36)^, foot washing^(15–17,22,24,26,30,32,35,36)^, proper use of footwear^(15–17,19,22,24,26,30,35,36)^, and the use of socks^(15–17,22,24,26,30,32,35,36)^ (10; 41.6% each). The least discussed topic in the videos was guidance on foot exercises^(16,26)^ (2;8.3%). Three studies^(21,28,33)^ (12.5%) did not mention the content of the video.

Figure 2 presents a summary of the content covered in the videos about foot self-care in the scoping review studies.

**Figure 2:**
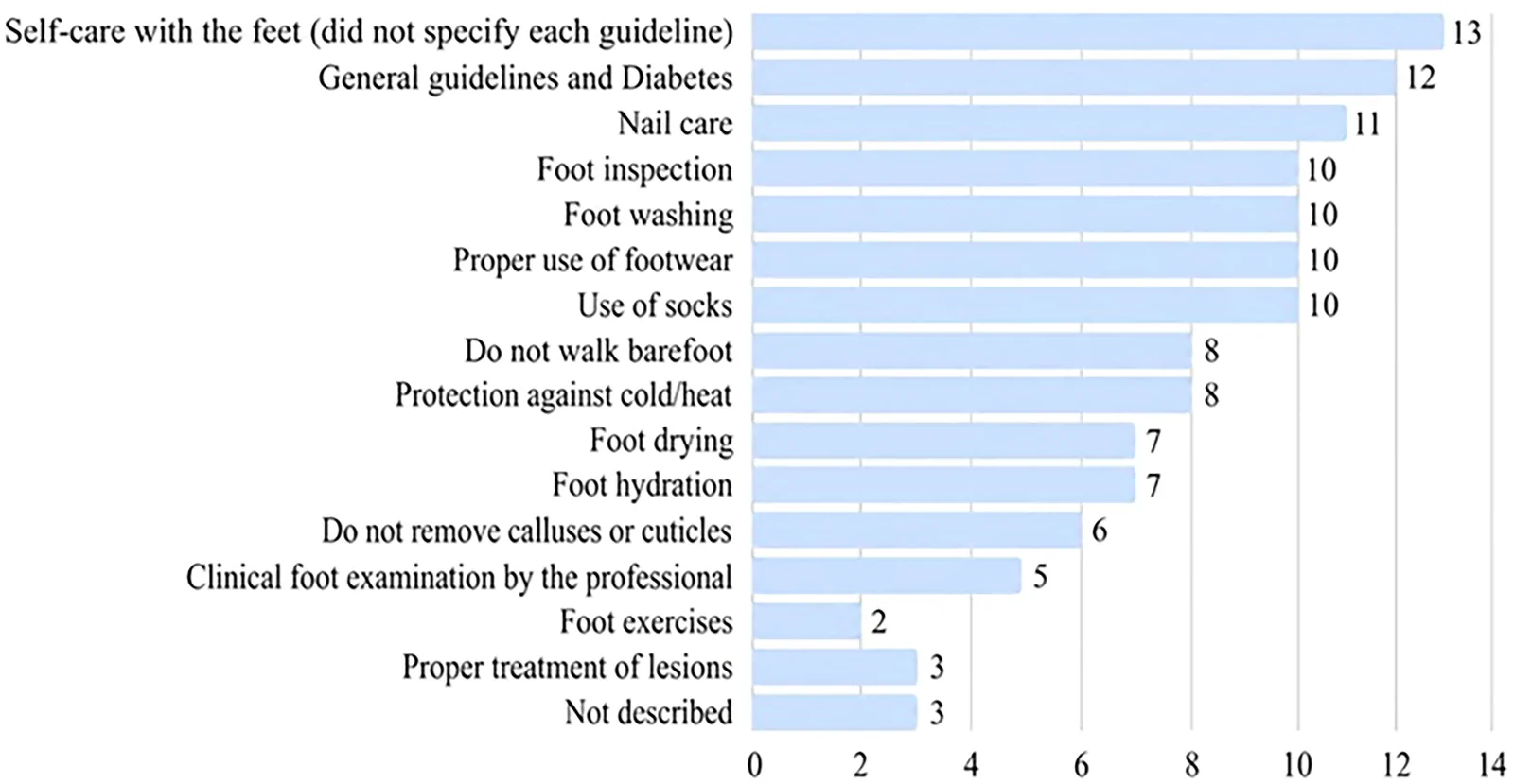
Summary of the content covered in the videos about foot self-care in the scoping review studies – Fortaleza, CE, Brazil, 2025.

**Source:** Authors (2025).

## DISCUSSION

The findings of this scoping review highlight the increasing use of educational videos as a strategy to support self-care in people with diabetes, especially in foot care, an essential aspect for the prevention of ulcers and amputations^(38,39)^. These resources have the potential to expand access to health information, promote patient empowerment, and strengthen educational initiatives in different healthcare settings.

Despite this potential, the included studies identified weaknesses related to the quality of the information provided. High heterogeneity, lack of clinical validation and standardized guidelines were observed regarding the content, duration, language, pedagogical strategies and audiovisual resources used, in addition to differences in the criteria employed for evaluating the materials. Studies that analyzed videos available on open platforms, such as YouTube, have demonstrated greater variability in scientific quality and less alignment with evidence-based recommendations when compared to materials produced by health institutions or academic groups^(40,41)^.

Furthermore, many videos presented incomplete or superficial information about fundamental preventive measures for foot self-care, including daily inspection, proper hygiene, footwear assessment, and early identification of injuries. It was observed that most of the materials did not fully comply with the recommendations of the *International Working Group on the Diabetic Foot* ^(1)^, limiting the educational potential and clinical applicability of these technologies.

The studies also highlighted important differences regarding the processes of video development and validation. Materials submitted to expert evaluation and user testing demonstrate greater clarity, organization, and suitability of information, promoting comprehension and applicability of educational guidelines^(30,42,43)^. Furthermore, it was observed that a large portion of the videos available on digital platforms do not undergo any validation process, which reinforces the urgency of incorporating clinical evaluation steps as a mandatory phase in the development of educational materials.

In this context, the need for standardized protocols for the production of educational videos in health is underscored. Although there are general recommendations on health education and digital communication, studies indicate a scarcity of systematized models to guide aspects such as scriptwriting, language definition, use of audiovisual resources, and adaptation of content to the needs of the target audience^(44,45)^. This lack of standardization hinders the comparison of studies, compromises the reproducibility of interventions, and limits the consolidation of evidence on the effectiveness of educational videos.

Another relevant aspect concerns the impact of inequality on access to educational technologies. Although videos can broaden the reach of health education initiatives, factors such as low socioeconomic status, limited internet access, and difficulties with digital literacy can restrict their use by more vulnerable populations^(46,47)^. Therefore, the incorporation of digital technologies in health education must be accompanied by public policies aimed at inclusion and expansion of access to technological tools.

In the Brazilian context, there has been progress in incorporating Digital Health as a structuring axis of the Brazilian Public Health System (SUS), especially through initiatives such as the National Digital Health Policy and *Conecte* SUS, which encourage the development and use of technological resources applied to healthcare^(48,49)^. However, the findings of this review demonstrate that the expansion of these tools must be coupled with the strengthening of mechanisms for evaluating the quality and detail of the information provided.

In this regard, the study by Smith et al.^(26)^, in evaluating videos on foot self-care for people with diabetes available on YouTube, identified that longer videos were significantly rated as helpful compared to those considered misleading or of little help. This result suggests that more detailed materials can promote greater scope in educational guidelines, although duration alone is not sufficient to guarantee scientific quality.

Beyond technical quality, the studies reinforce the importance of strengthening users’ critical thinking skills regarding health information disseminated in the technological environment. The widespread circulation of content without scientific curation can impair the identification of reliable sources, making it essential to develop educational strategies that promote digital literacy and the critical use of technologies in healthcare^(20,47,49)^.

Despite the limitations identified, educational videos remain promising tools to support foot self-care in people with diabetes, especially when used as a complement to in-person guidance provided by healthcare professionals. Its positive impact depends directly on the quality of the content, clinical validation, the use of appropriate teaching strategies, and increased access to technology.

The findings highlight the need to develop educational videos on foot self-care guidance that are in line with current guidelines, culturally adapted, and capable of meeting the different needs and sociocultural contexts of people with diabetes. Furthermore, they emphasize the importance of expanding access to digital technologies as a strategy to enhance health education initiatives, promote engagement in self-care, and strengthen the autonomy of people with diabetes in preventing foot-related complications.

The main limitations of this scoping review include the methodological heterogeneity of the included studies, which made direct comparison between results difficult, especially regarding the different formats, durations, and evaluation criteria of the educational videos. Moreover, many of the materials analyzed did not present sufficient information about validation processes or the theoretical basis used, limiting the assessment of content quality and credibility. The predominance of studies published in certain languages may have led to the underrepresentation of research originating from low- and middle-income countries, where the digital divide is more pronounced.

Finally, the database search may not have captured videos disseminated exclusively on non-indexed digital platforms, resulting in the potential loss of relevant material, suggesting that the findings should be interpreted with caution.

## CONCLUSION

The educational videos available and described in the literature represent a potentially valuable resource for strengthening foot self-care in people with diabetes. They have the potential to improve self-care and expand access to health education. However, its effectiveness depends on increased standardization, professional curation, and validation processes.

Investing in evidence-based audiovisual materials aligned with clinical guidelines is fundamental to improving the quality, safety, and applicability of these technologies in clinical practice and care to people with diabetes.

## DATA AVAILABILITY

All the data supporting the results of this study were published in the article itself.
