# Supplementary material for: Educational video technology for foot self-care in people with diabetes: a scoping review
Source: Rev Esc Enferm USP. 2026 Oct 30;60:e20260169. doi: 10.1590/1980-220X-REEUSP-2026-0169en (PMC13743178; doi:10.1590/1980-220X-REEUSP-2026-0169en)
Supplement: Translation

# Translation: Tecnologia educacional em vídeo para o autocuidado dos pés em pessoas com diabetes: revisão de escopo

**DOI:** 10.1590/1980-220X-REEUSP-2026-0169pt

**Authors:** Amelina de Brito Belchior, Florência Gamileira Nascimento, Victoria Maria Silva Leitão, Thiago Martins de Sousa, Lourival Veras de Oliveira, Cecília Lima de Souza, Sherida Karanini Paz de Oliveira

**Affiliations:** 1 Universidade Estadual do Ceará, Programa de Pós-Graduação em Cuidados Clínicos em Enfermagem e Saúde, Fortaleza, CE, Brasil.; 2 Universidade Estadual do Ceará, Centro de Ciências da Saúde, Departamento de Enfermagem, Fortaleza, CE, Brasil

**Keywords:** Complicações do Diabetes, Pé Diabético, Autocuidado, Filme e Vídeo Educativo, Tecnologia Biomédica

## Abstract

**Objetivo:**

Mapear, na literatura, estudos que abordem vídeos educacionais existentes sobre autocuidado com os pés de pessoas com diabetes.

**Método:**

Foi realizada uma revisão de escopo de acordo com as diretrizes do JBI. A busca foi realizada em nove bases de dados e repositórios, com critérios de inclusão definidos pela abordagem PCC (Participantes, Conceito e Contexto). A coleta de dados ocorreu entre março e junho de 2025.

**Resultados:**

Foram incluídos 24 estudos distribuídos em diferentes países, predominando o Brasil e Indonésia (6; 25,0% cada). Oito estudos (33,3%) descreveram o nome do vídeo. Sobre o conteúdo, destacam-se ações de autocuidado específicas com pés, como cuidado com as unhas (11;45,8%). Três estudos não citaram o conteúdo do vídeo.

**Conclusão:**

Os estudos disponíveis que abordam vídeos educacionais descritos na literatura representam um recurso potencialmente valioso para o fortalecimento do autocuidado com os pés em pessoas com diabetes. Apresentam potencial para fortalecer o autocuidado e ampliar o acesso à educação em saúde. Entretanto, sua efetividade depende de maior padronização, curadoria profissional e processos de validação.

## INTRODUÇÃO

O Diabetes Mellitus (DM) é uma das doenças crônicas que há muito tempo compõem um dos grandes problemas de saúde pública e está associada a diversas complicações que comprometem de maneira expressiva a qualidade de vida das pessoas. Entre essas complicações, destaca-se a doença do pé relacionada ao diabetes que gera impactos econômicos, sociais e psicológicos expressivos para os sistemas de saúde, paciente e família^(1)^. Estima-se que 131 milhões de pessoas no mundo apresentem complicações nos membros inferiores associadas ao diabetes, como neuropatia periférica e úlcera no pé relacionada ao diabetes, o que corresponde a 1,8% da população global^(2)^. Esse número, em constante crescimento, somado às dificuldades no manejo clínico, às limitações dos serviços de saúde e às desigualdades sociais contribui para taxas de amputação ainda consideradas inaceitáveis^(3)^.

Nesse contexto, o autocuidado desempenha papel essencial na prevenção e identificação precoce de complicações nos pés, sendo a educação estruturada, organizada e contínua fundamental para evitar úlceras e amputações relacionadas ao diabetes^(1)^. Medidas preventivas e ações educativas, inclusive por meio de intervenções tecnológicas, mostram-se imprescindíveis para reduzir riscos à saúde e fortalecer o autocuidado com os pés em pessoas com DM^(4,5)^.

As tecnologias têm evoluído rapidamente, tornando essencial que o setor saúde acompanhe essas inovações. Contudo, sua incorporação efetiva à prática clínica exige o desenvolvimento de materiais qualificados e a produção de evidências sistematizadas sobre sua aplicação^(6)^. Assim, materiais audiovisuais, por serem acessíveis e de amplo alcance, podem apoiar a prevenção de complicações, fortalecer o engajamento do paciente e complementar estratégias tradicionais de educação em saúde. Ao identificar e sistematizar essas iniciativas, potencializase a ampliação do uso de tecnologias digitais no cuidado ao diabetes, promovendo práticas mais eficazes, equitativas e sustentáveis^(7)^.

Nesse sentido, a utilização de vídeos educativos, como tecnologia educacional em saúde, é uma possibilidade concreta de apoio e de aprendizagem que visa compartilhar mensagens e orientações da forma mais clara e objetiva possível, para que o telespectador se sinta motivado e consiga reproduzir de forma eficiente as orientações prestadas^(8)^. Isso pode beneficiar a vida das pessoas com diabetes, facilitar a aprendizagem com instruções corretas de autocuidado com os pés, aprimorar e transformar as maneiras de cuidarem de si, tornando-os mais motivados e comprometidos com sua condição de saúde^(4)^.

Portanto, torna-se relevante desenvolver ações de orientação sobre o autocuidado com os pés para pessoas com diabetes por meio de tecnologias audiovisuais. Faz-se necessário, então, identificar os vídeos educacionais disponíveis na literatura científica, analisando suas características, potencialidades, conteúdos abordados e as lacunas ainda existentes.

Dessa forma, esse estudo tem como objetivo mapear, na literatura, os vídeos educacionais existentes sobre autocuidado com os pés de pessoas com diabetes.

## MÉTODO

### Tipo do Estudo

Trata-se de uma Revisão de escopo conduzida segundo as recomendações do *Joanna Briggs Institute* (JBI) e estruturada com base na *checklist Preferred Reporting Items for Systematic reviews and Meta-Analyses extension for Scoping Reviews* (PRISMA-ScR). O protocolo de pesquisa foi registrado na *Open Science Framework* (OSF) sob identificação: https://doi.org/10.17605/OSF.IO/BGTHK.

Foi desenvolvida em nove etapas: 1) Identificação do título e questão de pesquisa; 2) Definição dos critérios de elegibilidade de acordo com a estratégia PCC; 3) Seleção de fontes de evidência; 4) Definição da estratégia de busca; 5) Triagem e seleção das evidências; 6) Extração dos dados mediante formulário próprio; 7) Análise e síntese dos dados; 8) Apresentação dos resultados; e 9) Resumo das evidências em relação ao propósito da revisão, conclusões e implicações das descobertas^(9)^.

### Etapas do Estudo

#### Primeira Etapa – Identificação do Título e Questão de Pesquisa

Na primeira etapa, utilizou-se a estratégia PCC^(9)^, considerando: População – pessoas com diabetes; Conceito – vídeos educativos; e Contexto – autocuidado com os pés. Assim, formulou-se a seguinte questão de pesquisa: “Quais estudos que abordam vídeos educacionais sobre autocuidado com os pés de pessoas com diabetes estão disponíveis na literatura?”. A questão secundária foi: Quais os conteúdos abordados nos vídeos educativos para o autocuidado dos pés em pessoas com diabetes?

#### Segunda Etapa – Definição dos Critérios de Elegibilidade

A segunda etapa consistiu na definição dos critérios de inclusão e exclusão, conforme a estratégia PCC. Incluíram-se estudos com vídeos direcionados a pessoas de qualquer idade e tipo de diabetes que abordassem o autocuidado com os pés distribuídos em qualquer formato, tais como módulos on-line, aplicativos, redes sociais ou conteúdos armazenados em dispositivos móveis, em todos os idiomas e sem restrição temporal. Excluíram-se estudos com tecnologias exclusivamente para profissionais de saúde, que não especificaram o tipo de tecnologia utilizada, além de comentários, cartas ao editor, resumos, pôsteres de congressos, estudos de revisões, em razão da limitação de informações disponíveis. No entanto, foram analisadas suas referências para identificar e selecionar estudos que possam ser relevantes para inclusão.

#### Terceira Etapa – Seleção de Fontes de Evidência

A terceira etapa, seleção de fontes de evidência, foi realizada nas seguintes bases e repositórios de busca da área da saúde: *Medical Literature Analysis and Retrieval System Online* (MEDLINE) via PubMed, Literatura LatinoAmericana e do Caribe em Ciências da Saúde (LILACS) via Biblioteca Virtual em Saúde (BVS), *Web of Science, Scopus, Cumulative Index to Nursing and Allied Health Literature* (CINAHL) e Embase. A literatura cinzenta foi recuperada em quatros fontes: *Google Scholar*, Biblioteca Digital Brasileira de Teses e Dissertações (BDTD), Catálogo de Teses e Dissertações (CTD) da Coordenação de Aperfeiçoamento de Pessoal de Nível Superior (CAPES), *ProQuest Dissertations and Theses* (PQDT).

#### Quarta Etapa – Definição da Estratégia de Busca

Para definição da estratégia de busca, foram utilizados descritores, palavras-chaves e sinônimos de acordo com cada base de dados, por meio de pesquisa nos tesauros da saúde e dicionários reconhecidos na saúde, como *Medical Subject Headings* (MESH), Descritores em Ciências da Saúde (DECS) e EMTREE. Destaca-se que foram aplicados os filtros de teses e dissertações na plataforma Proquest.

Para a formulação de uma estratégia de busca sensível, utilizou-se a estratégia ECUS (extração, conversão, combinação, construção e uso)^(10)^, sendo utilizados os operadores booleanos OR e AND (Quadro 1).

**Quadro 1:** Estratégia de busca para a construção da revisão de escopo – Fortaleza, CE, Brasil, 2025^(10)^.

Objetivo/problema	Mapear vídeos educacionais voltados para o autocuidado com os pés de pessoas com diabetes			
	P	C	C		
Extração	Pessoas com diabetes mellitus	Vídeos educacionais	Autocuidado com os pés		
Conversão	Diabetes mellitus	educational video	Selfcare	diabetic foot
Combinação	Diabetes mellitus, Diabetic Neuropathies, diabetes, diabetic, diabetic patient	Webcasts, video, educational video, infographics, video technology, streaming video, video animation	Selfcare, self-care, self-management;	diabetic foot, Diabetic foot, Diabetic Feet, Ulcer Diabetic foot, diabetic foot wound, diabetic foot ulcer, diabetic foot sore
Construção	(“diabetes mellitus” OR “Diabetic Neuropathies” OR diabetes OR diabetic OR “diabetic patient”)	(Webcasts, OR vídeo OR “educational video” OR infographics OR “video technology” OR “streaming video” OR “video animation”)	(Selfcare OR self-care OR self-management OR selfmanagement) AND (“diabetic foot” OR Diabetic foot” OR “Diabetic Feet” OR “Ulcer Diabetic foot” OR “diabetic foot wound” OR “diabetic foot ulcer” OR” diabetic foot sore”)	
#Uso 1	(“diabetes mellitus” OR “Diabetic Neuropathies” OR diabetes OR diabetic OR “diabetic patient”) AND (Webcasts, OR vídeo OR “educational video” OR infographics OR “vídeo technology” OR “streaming video” OR “video animation”) AND (Selfcare OR self-care OR self-management OR selfmanagement) AND (“diabetic foot” OR Diabetic foot” OR “Diabetic Feet” OR “Ulcer Diabetic foot” OR “diabetic foot wound” OR “diabetic foot ulcer” OR “diabetic foot sore”).			
#Uso 2	(“diabetes mellitus OR “neuropatias diabéticas” OR diabetes OR diabético) AND (Webcast OR vídeos OR “vídeos educacionais” OR Infograficos OR “animação em vídeo” OR “tecnologia em vídeo”) AND (autocuidado OR autogerenciamento) AND (“pé diabético” OR “pés diabéticos” OR “úlcera do pé diabético”).			

#### Quinta Etapa – Triagem e Seleção das Evidências

A seleção dos estudos foi realizada pelo *software Rayyan* que permite a triagem de forma cega e independente entre os revisores, procedendo a análise de títulos, resumos e leitura na íntegra dos estudos encontrados na literatura, removendo as duplicatas.

#### Sexta Etapa – Extração dos Dados Mediante Formulário Próprio

Na sexta etapa, extração dos dados mediante formulário próprio, dois examinadores participaram da elegibilidade dos estudos de forma independente, utilizando o software *Endnote web* (http://www.myendnoteweb.com/EndNoteWeb.html) e planilhas do Excel para gerenciamento dos estudos. Inicialmente, os examinadores realizaram uma triagem a partir da leitura dos títulos e resumos. Após reunião de consenso, confirmou-se a seleção dos artigos, justificando-se a exclusão conforme os critérios estabelecidos. Os estudos foram lidos na íntegra, e, em seguida, as referências foram analisadas para inclusão de novos estudos. Essa etapa ocorreu entre março e junho de 2025.

A extração e o gerenciamento dos dados foram efetuados por meio de um mapeamento, contendo informações de caracterização como autoria, ano, país, título objetivo, tipo de estudo, idioma, nível de evidência baseada na classificação hierárquica de Melnyk e Fineout-Overholt^(11)^ e características dos vídeos.

#### Sétima Etapa – Análise e Síntese dos Dados

Na sétima etapa, análise e síntese dos dados, realizou-se concordância entre os revisores pela análise do coeficiente de *Kappa de Cohen*, adotando-se os seguintes parâmetros: 0–0,20, nenhum; 0,21–0,39, mínimo; 0,40–0,59, fraco; 0,60–0,79, moderado; 0,80–0,90 forte; acima de 90, quase perfeito^(12)^.

Na extração dos dados, elaborou-se um resumo crítico, sintetizando todas essas informações. É importante salientar ainda que o nível de evidência segundo essa referência é determinado de acordo com as características metodológicas dos estudos; quanto maior o nível de evidência, melhor representação de estudos com maior grau de confiabilidade para a tomada de decisão. Ademais, consideraram-se, ainda, características da própria produção textual dos estudos, como nome do vídeo criado, caracterização da tecnologia, duração, público-alvo, linguagem utilizada.

Os resultados foram apresentados por meio de materiais gráficos como quadros, tabelas, figuras e analisados à luz da literatura atualizada pertinente.

A oitava etapa, apresentação dos resultados, e a nona, resumo das evidências em relação ao propósito da revisão, conclusões e implicações das descobertas, estão dispostas na sequência.

### Aspectos Éticos

Para a realização deste estudo, respeitaram-se todos os preceitos éticos. Todos os autores dos artigos analisados foram referenciados adequadamente, conforme a Lei de Direitos Autorais nº 9.610, de 19 de fevereiro de 1998^(13)^. Os dados e informações das pesquisas foram apresentados de forma fidedigna.

Durante a preparação deste artigo, os autores não utilizaram ferramentas de geração de texto e/ou Inteligência Artificial (IA).

## RESULTADOS

A busca identificou 1.906 potencialmente relevantes, dos quais 653 foram removidos por duplicidade. Os 1.253 estudos restantes foram selecionados por título e resumo. Desse total, 50 estudos foram incluídos para análise de texto completo por dois revisores independentes. Em seguida, após a leitura na íntegra, 31 estudos foram excluídos por não responderem à questão da pesquisa, restando 19 estudos.

A partir da consulta nas listas de referências, foram identificados cinco estudos para compor a amostra, resultando em 24 estudos incluídos nessa revisão de escopo. A Figura 1 evidencia o processo de seleção.

**Figura 1:** Fluxograma PRISMA-ScR de seleção dos estudos da revisão de escopo – Fortaleza, CE, Brasil, 2025.

**Fonte:** Dados da pesquisa (2025), adaptado de Page et al.^(14)^.

Os estudos incluídos estão distribuídos em diferentes países, predominando o Brasil^(4,15,16,17,18,19)^ e Indonésia^(20,21,22,23,24,25)^ (6;25,0% cada), seguidos dos Estados Unidos^(26,27,28)^, (3;12,5%), Turquia(29,30) (2;8,3%), Portugal^(29)^, Tunísia^(30)^, Índia^(31)^, Marrocos^(32)^, Holanda^(33)^ (1;4,1% cada). Dois estudos foram realizados em dois países: Arábia Saudita e Reino Unido^(34)^ e Arábia Saudita e Canadá^(35)^ (1;4,2% cada).

O estudo mais antigo foi publicado em 1987^(33)^, (1;4,1%), com maior prevalência nos anos de 2022^(17,18,19,34,36)^ e 2023^(15,16,20,29,30)^ (5;20,8 cada). Outros tiveram menor frequência de publicação, em 2011^(27,28)^, 2019^(24,26)^ (2;8,3% cada), 1987^(33)^, 2014^(25)^, 2015^(35)^, 2017^(32)^, 2021^(21)^, 2024^(4)^ (1;4,1% cada).

O tipo de publicação mais frequente foi artigo^(4,17,19,20,21,22,23,24,25,26,27,28,29,30,31,32,33,34,35,36,37)^ (21;87,5%) seguido de dissertação^(15,16,18)^ (3;12,5%), publicados no idioma inglês^(24,26,27,28,29,31,32,33,34,35,36,37)^ (12;50,0%), português ^(4,15,16,17,18,19)^ (6;25,0%), indonésio^(20,21,22,23,25)^ (5;20,8%) e francês^(30)^ (1;4,1%).

Com relação ao método, destacam-se estudos quase-experimentais^(20,21,22,23,24,25,28,30)^(8;33,3%), seguidos de metodológico^(15,16,17,18,19)^ e Ensaio Clínico Randomizado^(27,29,31,32,37)^ (5;20,8% cada). Quatro artigos^(26,34,35,36)^ (16,6%) avaliaram os vídeos sobre autocuidado com os pés como fonte de informações disponíveis na plataforma *Youtube* e um artigo^(29)^ (4,1%) trata de um protocolo de um Ensaio Clínico Randomizado.

Os estudos encontrados apresentaram nível de evidência II^(27,29,31,32,37)^ (5;20,8%), III^(20,21,22,23,24,25,26,28,30)^ (9;37,5%) e VI^(4,15,16,17,18,19,33,34,35,36)^ (10;41,6%) com maior frequência.

O Quadro 2 apresenta a caracterização dos estudos incluídos na revisão, quanto à autoria, ano de publicação, país, título, objetivos, amostra, idioma, método e nível de evidência.

**Quadro 2:** Caracterização dos estudos selecionados para a revisão de escopo – Fortaleza, CE, Brasil, 2025.

ID	Autor (ano)/país	Título	Objetivo	Amostra	Idioma	Método/nível de evidência
E1	Andrade et al. (2024)^(4)^. Brasil	Percepção dos usuários com Diabetes sobre o Autocuidado com os Pés: Uma análise qualitativa	Avaliar a percepção sobre o uso de vídeos educativos para o autocuidado com os pés em usuários com DM2 na Atenção Primária no estado do Amazonas.	20 pessoas com DM2.	Português	Estudo qualitativo (Artigo)/VI
E2	Ferreira et al. (2023)^(29)^. Portugal	Educational Interventions on Diabetic Foot Self-Care: A Study Protocol for a Pragmatic Randomized Controlled Trial	Analisar o impacto de diferentes estratégias educacionais – um vídeo instrutivo.	54 pessoas com DM e úlcera nos pés, 18 em cada grupo	Inglês	Ensaio clínico randomizado (Artigo)/II
E3	Octaviana e Hati (2023)^(20)^. Indonésia	Analisis Pengaruh Edukasi Melalui Media Video terhadap Tingkat Pengetahuan Pasien Diabetes Melitus Tentang Perawatan Kaki di Puskesmas	Analisar a eficácia da educação sobre cuidados com os pés por meio de vídeos no nível de conhecimento de pacientes com diabetes mellitus.	60 pessoas com DM	Indonésio	Estudo Quase-experimental (Artigo)/III
E4	Oliveira (2023)^(15)^. Brasil	Construção e validação de vídeo educativo para prevenção do pé diabético centrado no apoio social	Construir e validar um vídeo educativo para a prevenção do pé diabético centrado no apoio social da pessoa com DM.	26 especialistas (profissionais de saúde)	Português	Estudo metodológico (Dissertação)/VI
E5	Yazidi et al. (2023)^(30)^. Tunísia	Place des supports vidéo disponibles en ligne dans l’éducation thérapeutique des diabétiques: étude évaluative chez une population de diabétiques à haut risque podologique	Avaliar o impacto de um vídeo educativo online na melhoria do conhecimento de pacientes diabéticos com alto risco de problemas nos pés relativamente à prevenção de lesões nos pés.	40 pessoas com diabetes com alto risco nos pés.	Francês	Estudo quase-experimental (Artigo)/III
E6	Siqueira (2023)^(16)^. Brasil	Vídeo Educativo: atividades de autocuidado para prevenção de complicações nos pés de pessoas idosas com diabetes	Construir um vídeo educativo sobre as atividades de autocuidado para prevenção de complicações nos pés da pessoa idosa com diabetes.	9 especialistas (enfermeiros)	Português	Estudo metodológico (Dissertação)/VI
E7	Almaqhawi et al. (2023)^(34)^. Arábia Saudita e Reino Unido	Evaluation of quality of diabetic foot examination on YouTube	Avaliar a qualidade educacional e a confiabilidade de vídeos disponíveis publicamente no YouTube sobre exames dos pés em pessoas com diabetes, a fim de verificar se o YouTube é ou não uma fonte confiável de informações de saúde para pacientes.	100 vídeos do YouTube	Inglês	Estudo descritivo (Artigo)/VI
E8	Doğan e Pehlivan (2022)^(36)^. Turquia	Diabetic foot care training and the presence of nurses in Turkish YouTube videos	Analisar o conteúdo de vídeos turcos do YouTube como fonte de informação sobre treinamento em cuidados com os pés de pacientes diabéticos e a presença de enfermeiros nesses vídeos.	87 vídeos do YouTube	Inglês	Estudo descritivo (Artigo)/VI
E9	Menezes et al. (2022)^(17)^. Brasil	Produção e validação do curta-metragem Pés que te quero®: tecnologia educacional para pessoas com diabetes	Descrever a produção e validação de tecnologia educacional do tipo curta-metragem para prevenção de úlceras do pé diabético.	31 especialistas da enfermagem, 6 da comunicação e 15 pessoas com DM2	Português	Estudo metodológico (Artigo)/VI
E10	Nascimento (2022)^(18)^. Brasil	Construção e validação do vídeo cordel “ô pé arretado” como tecnologia educativa para o pé diabético.	Construir e validar uma literatura em cordel com apresentação em vídeo como tecnologia educacional para a prevenção do pé diabético	8 acadêmicas de Enfermagem e 6 especialistas para a Validação de Conteúdo e Aparência e quatro pessoas com DM para avaliar a usabilidade	Português	Estudo metodológico (Dissertação)/VI
E11	Meneses et al. (2022)^(19)^. Brasil	Desenvolvimento e validação de vídeo educativo sobre cuidados podiátricos para prevenção de úlceras em idosos com diabetes	Desenvolver e validar um vídeo educativo sobre cuidados podiátricos para prevenção do pé diabético em idosos.	10 especialistas.	Português	Estudo metodológico (Artigo)/VI
E12	Wicahyani et al. (2021)^(21)^. Indonésia	Aplikasi Edukasi Berbasis Video Untuk Meningkatkan Dukungan Keluarga Mencegah Komplikasi Kaki Diabetes	Investigar a eficácia da educação baseada em vídeo no aumento do apoio familiar para a prevenção de complicações nos pés de pacientes com diabetes mellitus tipo 2.	80 pessoas com diabetes e seus familiares.	Indonésio	Estudo quase-experimental (Artigo)/III
E13	Hamzah et al. (2020)^(22)^. Indonésia	The effectiveness of foot care education video in Makassar language to increase patients’ knowledge related foot care in community	Determinar a eficácia de um vídeo educativo sobre cuidados com os pés, em língua Makassar, na melhoria do conhecimento de pacientes com DM sobre o tema.	30 pessoas com DM sem úlceras nos pés	Indonésio	Estudo quase-experimental (Artigo)/III
E14	Dinçer e Bahçecik (2020)^(37)^. Turquia	The effect of a mobile application on the foot care of individuals with type 2 diabetes: A randomised controlled study	Desenvolver um aplicativo móvel de educação sobre cuidados com os pés em diabéticos (M-DFCE) com suporte de animações para uso por indivíduos com diabetes tipo 2 e identificar seus efeitos sobre o conhecimento, a autoeficácia e o comportamento de cuidados com os pés.	130 pessoas com diabetes (65 no grupo experimental e 65 no grupo controle).	Inglês	Ensaio clínico randomizado (Artigo)/II
E15	Nurjanna et al. (2020)^(23)^. Indonésia	Perbandingan pengetahuan self efficacy perawatan kaki pada pasien Diabetes Melitus tipe II setelah menggunakan video edukasi di puskesmas paccerakkang kota makassar	Comparar o conhecimento sobre autoconfiança em cuidados com os pés em pacientes com Diabetes Mellitus tipo 2 após a utilização de vídeos educativos no Centro de Saúde Comunitária de Paccerakkang, na cidade de Makassar.	20 pacientes com DM	Indonésio	Estudo quase-experimental (Artigo)/III
E16	Abrar et al. (2020)^(24)^. Indonésia	Development and evaluation educational videos of diabetic foot care in traditional languages to enhance knowledge of patients diagnosed with diabetes and risk for diabetic foot ulcers	Desenvolver um vídeo educativo sobre cuidados com os pés diabéticos nas línguas tradicionais (buginese e macassarese) e avaliar o efeito desses vídeos no conhecimento do paciente.	7 enfermeiros especialistas para desenvolvimento do conteúdo do vídeo, três para a validade de conteúdo e 20 pessoas de cada grupo étnico com DM com risco de úlcera nos pés	Inglês	Estudo quase-experimental (Artigo)/III
E17	Smith et al. (2019)^(26)^. EUA	Analysis of YouTube as a Source of Information for Diabetic Foot Care	Alertar os médicos sobre a qualidade das informações sobre cuidados com os pés diabéticos no YouTube e estabelecer se o YouTube é uma fonte útil de informações para pacientes com problemas nos pés diabéticos.	87 vídeos do Youtube	Inglês	Estudo descritivo (Artigo)/VI
E18	Rahaman et al. (2018)^(31)^. Índia	Effectiveness of a Patient Education Module on Diabetic Foot Care in Outpatient Setting: An Open-label Randomized Controlled Study	Desenvolver um módulo educativo para pacientes sobre cuidados com os pés diabéticos e a avaliar sua eficácia no conhecimento e na prática de cuidados com os pés em pacientes com diabetes.	127 (participaram de todas as etapas: 101 pacientes com DM1 e DM2 GI: 63 (participaram de todas as etapas: 51). GC: 64 (participaram de todas as etapas: 50)	Inglês	Ensaio clínico randomizado (Artigo)/II
E19	Adarmouch et al. (2017)^(32)^. Marrocos	Short-term effectiveness of a culturally tailored educational intervention on foot self-care among type 2 diabetes patients in Morocco	Avaliar a eficácia de uma intervenção de educação para o autocuidado adaptada culturalmente às práticas de autogestão do cuidado dos pés em pacientes com diabetes tipo 2 e identificar os fatores associados à variação dessas práticas.	199 pessoas com DM foram recrutadas e participaram da primeira e 133 completaram a segunda avaliação	Inglês	Ensaio clínico randomizado (Artigo)/II
E20	Abedin et al. (2015)^(35)^. Arábia Saudita e Canadá	YouTube as a source of useful information on diabetes foot care on diabetes foot care	Analisar o uso do YouTube como fonte eficaz de informação sobre cuidados com os pés diabéticos.	89 vídeos do Youtube	Inglês	Pesquisa documental (Artigo)/VI
E21	Setyorini et al. (2014)^(25)^. Indonésia	Edukasi perawatan kaki melalui media guidance motion picture dan leaflet terhadap perilaku perawatan kaki non ulkus pada penderita Diabetes Melitus Tipe 2 di Puskesmas Loceret	Avaliar eficácia da educação sobre cuidados com os pés por meio de recursos audiovisuais e folhetos sobre cuidados com os pés sem úlceras em pacientes com DM2 no Centro de Saúde Comunitária de Loceret.	30 pacientes com DM2	Indonésio	Estudo quase-experimental (Artigo)/III
E22	Grady et al. (2011)^(27)^ EUA	Using message framing to achieve long-term behavioral changes in persons with diabetes	Examinar o impacto da forma como a informação é apresentada em um programa educacional sobre cuidados adequados com os pés e sua importância na prevenção de complicações do diabetes, considerando mudanças em longo prazo no conhecimento, nas atitudes e no comportamento em relação aos cuidados com os pés.	155 pessoas com diabetes há 5 anos ou mais e sem histórico de problemas nos pés.	Inglês	Ensaio clínico randomizado (Artigo)/II
E23	Gravely et al. (2011)^(28)^. EUA	Comparison of three types of diabetic foot ulcer education plans to determine patient recall of education	Comparar o material educacional escrito atualmente, usado com um vídeo educativo e uma combinação de material escrito e vídeo para determinar a lembrança do paciente sobre a educação.	30 pessoas com DM	Inglês	Estudo quase-experimental (Artigo)/III
E24	Bakker (1987)^(33)^. Holanda	Video Education on Foot-Care	Educar pacientes e funcionários e verificar se um hospital com a assistência de um diretor de vídeo profissional seria capaz de produzir um vídeo de qualidade com um orçamento reduzido.	40 pacientes com DM	Inglês	Estudo descritivo observacional (Artigo)/VI

Legenda: DM: diabetes mellitus; DM2: diabetes mellitus tipo 2; DM1: diabetes mellitus tipo1; GC: grupo controle; GI: grupo intervenção; M-DFCE: Mobile Diabetic Foot Care Education.

Fonte: Autores (2025).

Observou-se que em oito estudos (33,3%) estavam descritos os nomes dos vídeos: “Verdadeiro ou Falso? e Diabetes e Cuidados diários com os pés”^(4)^, “Pés de quem Cuido”^(15)^, “Autocuidado com os pés de idosos com diabetes”^(16)^, “Pés que te quero”®(17), “Ô pé arretado”^(18)^, “No rastro da prevenção: Uma narrativa sobre o diabetes e os cuidados com os pés na pessoa idosa”^(19)^, “*Guidance Motion Picture”* (GMP)^(25)^ e o *Mobile Diabetic Foot Care Education* (M-DFCE)^(37)^, que contém vídeos de animação sobre orientações de autocuidado com os pés.

Entre os vídeos cujo tempo foi informado^(15,16,17,18,19,21,22,24,26,27,29,30,31,33 34,35,36,37)^ (18;75,0%), a duração variou de 2 minutos e 51 segundos^(18)^ até 28^(33)^ e 30 minutos (37). Predominaram vídeos com duração entre 3 a 10 minutos^(16,18,19,22,24,26,27,29,30,31,37)^ (11;45,8%). Ressalta-se que dos quatros estudos (16,6%) que avaliaram os vídeos do *Youtube* ^(23,31,34,36)^, dois (8,3%) trouxeram a média do tempo dos vídeos, 6,8 ± 6,6’^(34)^ e 8,08±13,27’^(36)^ e dois (8,3%), o tempo máximo dos vídeos disponíveis na plataforma, até 10’^(26)^ e até 30’^(35)^.

Quanto à intervenção realizada nos estudos, houve prevalência do delineamento pré e pós-teste^(20,21,22,23,24,28,30,32,33)^ (9;37,5%) com aplicação de questionários para avaliação antes e após a exposição ao vídeo educativo. Outros estudos compararam o uso de vídeos com materiais impressos, orientações tradicionais ou tratamento padrão^(25,29,31,37)^ (4;16,6%), enquanto alguns se concentraram na validação de conteúdo e aparência dos vídeos por especialistas^(15,16,19)^ (3;12,5%) e por especialistas e público-alvo^(17,18)^ (2;8,3%). Também foram identificados estudos de análise de qualidade e utilidade de vídeos disponíveis online^(26,34,35,36)^ (4;16,6%). Um estudo^(4)^ (4,1%) aplicou um questionário após o paciente assistir dois vídeos educativos, com o intuito de entender a percepção dos usuários sobre os cuidados com os pés antes e depois de assistirem aos vídeos. Outro estudo^(27)^ (4,1%) expôs dois vídeos a grupos diferentes, um com mensagens de prevenção com foco nos ganhos, outro nas perdas, para promover comportamentos que mantêm a saúde em pessoas com diabetes. Um artigo^(29)^ (4,1%) trouxe um protocolo de estudo sobre o impacto de duas estratégias educativas diferentes, sem informação dos resultados.

Os principais resultados relatados abrangem aumento do conhecimento sobre cuidado com os pés^(4,20,22,23,24,25,28,30,31,33,37)^ (11;45,8%), melhora nas práticas de autocuidado^(4,20,32,33,37)^ (5;20,8%) e mudanças positivas de atitude^(4,21)^ (2;8,3%).

Um estudo^(21)^ (4,1%) investigou a eficácia de um vídeo para a prevenção de complicações nos pés de pacientes com DM2 no aumento do apoio familiar e revelou que vídeos educacionais sobre o exame dos pés de pacientes com diabetes podem modificar atitudes e aumentar o apoio familiar na realização dos cuidados com os pés desses pacientes.

Sobre os estudos que avaliaram vídeos online, os resultados descrevem a proporção de vídeos considerados úteis, moderadamente úteis ou enganosos, bem como as características das fontes produtoras do conteúdo^(26,34,35,36)^ (4;16,6%).

O Quadro 3 apresenta a sumarização dos principais conteúdos abordados, intervenção e principais resultados dos estudos incluídos na revisão.

**Quadro 3:** Principais conteúdos abordados, intervenção e resultados dos estudos – Fortaleza, CE, Brasil, 2025.

ID	Nome	Tempo	Intervenção	Principais resultados
E1^(4)^	Verdadeiro ou Falso? e Diabetes e Cuidados diários com os pés	Não descrito	Os participantes foram visitados em suas casas por um pesquisador. Durante as entrevistas, os participantes estavam próximos a um parente ou conhecido. As perguntas foram estruturadas para entender a percepção dos usuários sobre os cuidados com os pés antes e depois de assistirem aos vídeos.	Surgiram três categorias e duas subcategorias temáticas: 1) ‘conhecimento prévio’ com as subcategorias ‘fragilidade nas orientações profissionais’ e ‘os vídeos como instrumentos para relembrar’; 2) ‘assimilação da linguagem e formato audiovisual’; 3) ‘possibilidades de impacto sobre o autocuidado
E2^(29)^	Não descrito	~6’	Vídeo instrutivo (GI 1) comparado com um folheto informativo sobre cuidados com os pés com leitura guiada em tempo real (GI 2) e cuidados padrão (GC) – na adesão e no conhecimento sobre cuidados com os pés de pessoas com diabetes e na percepção dos pacientes sobre a saúde dos seus pés, ao longo do tempo.	Não descrito.
E3^(20)^	Não descrito	Não descrito	Houve aplicação de pré-teste e pós-teste. No GC, nenhum vídeo educativo foi exibido e um questionário sobre cuidados com os pés foi aplicado nas semanas 1 e 4. Já no GI, o questionário foi aplicado a partir da semana 1, e nas semanas 2 e 3, um vídeo educativo sobre cuidados com os pés foi exibido. Na semana 4, o questionário foi aplicado novamente.	Os resultados do teste de Mann-Whitney antes da exibição do vídeo foram de 0,957, não havendo diferença significativa entre os GI e GC. Já após a exibição, o resultado foi de 0,000, indicando diferença significativa entre os GI e GC. O uso de vídeos educativos na educação aumentou significativamente o conhecimento e autocuidado com os pés em pacientes com DM2.
E4^(15)^	“Pés de quem cuido”	17’	Não foi realizado	Na análise dos IVC de todas as subcategorias foram considerados válidos e confiáveis. A terceira versão do roteiro obteve IVC de 0,94. Entre as principais considerações feitas pelos juízes no processo de validação, destaca-se a adequação da linguagem dos personagens e redução dos textos (falas dos personagens).
E5^(30)^	Não descrito	3’31”	Foi aplicado um questionário sobre conhecimento teórico e prático sobre a prevenção de lesões nos pés em pessoas com DM. Em seguida, os pacientes assistiram ao vídeo. Uma reavaliação do conhecimento, utilizando o mesmo questionário, foi realizada no mesmo dia.	Mais de 90% das pessoas declararam estar “satisfeitas” ou “muito satisfeitas” com o vídeo educativo. Observou-se melhora significativa no conhecimento sobre a prevenção de lesões nos pés em pessoas com DM. O impacto do vídeo na redução da frequência de úlceras crônicas e amputações ainda precisa ser determinado.
E6^(16)^	Autocuidado com os pés de idosos com diabetes	6’5”	Não foi realizado	Primeiramente, analisaram-se 16 artigos que apontaram evidências na prevenção de complicações nos pés de pessoas idosas com diabetes; após isso, utilizou-se como arcabouço para produção do vídeo educativo. Após avaliação de nove especialistas, obteve-se IVC geral igual a 0,90.
E7^(34)^	Não se aplica	Média de 6,8 ± 6,6’	Não se aplica	A categoria de conteúdo mais frequente foi exame dos pés (58 vídeos), seguida por informações específicas sobre a doença (53 vídeos). A fonte de upload mais comum foi de médicos (28%) e a menos comum, de pacientes (1%).
E8^(36)^	Não descrito	Média de 8,08 ± 13,27’	Não se aplica	Dos vídeos avaliados, 8% foram classificados como muito úteis, 33,3% moderadamente úteis, 51,7% um tanto úteis e 6,9% não úteis. Destaca-se que 4,6% continham informações enganosas. O instrutor era enfermeiro em apenas 12,6% desses vídeos.
E9^(17)^	“Pés que te quero”	24’7”	Não foi realizado	O roteiro do vídeo apresentou IVC total de 0,95, com confiabilidade interna de 0,849 conferido pelo alfa de Cronbach, proficientes da comunicação validaram com IVC de 0,97 e público-alvo com 0,95 (clareza) e 0,97 (relevância).
E10^(18)^	“Ô Pé arretado”	2’51”	Não foi realizado	Na validação com juízes técnicos verificou-se 100% de concordância na simplicidade, clareza e relevância do conteúdo e um CVCt de 1,0 do roteiro do vídeo cordel. Além de 100% de concordância por parte dos juízes da prática clínica quanto a simplicidade, clareza e relevância do conteúdo e aparência do vídeo-cordel com concordância de adequação do material educativo para o autocuidado na prevenção do pé diabético, assim como complicações da doença.
E11^(19)^	“No rastro da prevenção: Uma narrativa sobre o diabetes e os cuidados com os pés na pessoa idosa”	4’33”	Não foi realizado	Observou-se concordância unânime entre os especialistas para a maioria dos critérios. Ainda assim, conforme o critério adotado para validação do conteúdo do vídeo que foi o IVC global, calculado pela média dos IVCs de cada item, não se mostrou necessária nenhuma alteração no conteúdo do vídeo proposto, considerando-se o conteúdo do vídeo como válido com 99,38% de concordância entre os juízes (IVC = 0,99).
E12^(21)^	Não descrito	12’	Aplicou-se um pré-teste via WhatsApp e, em seguida, o vídeo educativo foi enviado. Após assistirem o vídeo, os participantes realizaram o exame nos pés de forma independente e enviaram um vídeo com a gravação do exame que realizaram. O pós-teste foi realizado online uma semana após o pré-teste, utilizando um formulário do Google.	Vídeos educacionais sobre o exame dos pés de pacientes com diabetes podem modificar atitudes e aumentar o apoio familiar na realização dos cuidados com os pés desses pacientes. Com base nos resultados da análise estatística de Wilcoxon, o valor de p foi de 0,009 (p < 0,05), com uma pontuação mediana de apoio familiar total de 77 antes e 81,6 após a intervenção.
E13^(22)^	Não descrito	5’15”	Aplicou-se um pré-teste e pós-teste em um único grupo antes e após a visualização do vídeo da pesquisa de Abrar et al. (2020).	Os resultados revelaram que houve aumento do bom conhecimento (36,7%–90%) e diminuição do conhecimento moderado (56,7%–10%) e do conhecimento insuficiente (6,7% para 0%).
E14^(37)^	*Mobile Diabetic Foot Care Education* (M-DFCE)	6 seções, cada uma com duração aproximada de 1’30’’	GI: recebeu orientações sobre cuidados com os pés de pacientes diabéticos por meio de um aplicativo de celular. GC: recebeu orientações sobre cuidados com os pés diabéticos de acordo com as diretrizes clínicas do hospital. O vídeo foi apresentado uma vez no início da sessão de Educação em Saúde e um acompanhamento foi realizado um mês após.	Houve um aumento significativo no nível de conhecimento dos pacientes do GI sobre cuidados com os pés (p = 0,001). Não houve aumento significativo na pontuação de conhecimento do GC entre a primeira e a última avaliação (p = 0,905). Não houve diferença entre os grupos na avaliação inicial, porém, os participantes do GI que receberam M-DFCE com suporte de animação apresentaram níveis significativamente mais elevados de conhecimento, autoeficácia e comportamento de cuidado com os pés do que o GC.
E15^(23)^	Sem Informação	Não descrito	Aplicação de Vídeo educativo sobre cuidados com os pés (pré e pós-teste)	O conhecimento da amostra sobre cuidados com os pés antes do vídeo educativo teve média de 6,40 ± 1,789, mediana de 7,0 e a pontuação de conhecimento após o vídeo alcançou média de 10,70 ± 0,571, mediana de 11,00 (p = 0,000).
E16^(24)^	Não descrito	>6’	O pré-teste foi distribuído aos participantes antes da exibição do vídeo. O vídeo foi reproduzido usando projetor de imagem por menos de 6’. O pós-teste foi realizado com os mesmos questionários.	O conhecimento dos participantes sobre cuidados com os pés aumentou significativamente depois de assistir aos vídeos educativos em ambos os idiomas, Makassarese (p = 0,001) e o idioma bugisês (p = 0,001).
E17^(26)^	Não descrito	>10’	Não se aplica	Dos 87 vídeos incluídos, 56 (64,4%) foram classificados como úteis e 31 (35,6%) como não úteis/ enganosos. Observou-se uma diferença significativa na duração média dos vídeos úteis em comparação com os vídeos não úteis/enganosos (3,33 versus 1,73 min; p < 0,0001). Não houve diferença significativa em termos de métricas de popularidade (curtidas, visualizações, inscrições, etc.) entre os vídeos úteis e não úteis/enganosos.
E18^(31)^	Sem informação	9’	O GI recebeu no módulo educativo sobre cuidados com os pés, um vídeo audiovisual. O GC recebeu atendimento de rotina.	As pontuações de conhecimento no GI na primeira, segunda e terceira consultas foram de 9,8 ± 1,8, 10,2 ± 1,6 e 11,0 ± 1,7, respectivamente. As pontuações de conhecimento no GC na primeira, segunda e terceira consultas foram de 9,9 ± 1,7, 9,8 ± 1,6 e 10,0 ± 1,8, respectivamente. A mudança na pontuação de conhecimento foi estatisticamente significativa (p < 0,001) na terceira consulta em comparação com a primeira no grupo de intervenção, mas não no GC (p = 0,62). A pontuação de prática também melhorou significativamente (p < 0,001) no GI na segunda consulta, mas não no GC.
E19^(32)^	Não descrito	Sem informação	A intervenção consistiu em uma discussão interativa em grupo utilizando diferentes materiais: um vídeo narrativo, uma apresentação em PowerPoint e um guia impresso. Foram realizados pré e pós-teste.	A pontuação média de cuidados com os pés aumentou de 3,5 ± 2,9 dias para 5,9 ± 1,8 dias em um mês após a intervenção (variação média de 2,4 ± 3,1 dias; p < 0,001). Uma variação favorável foi encontrada em 75 (37,7%) participantes.
E20^(35)^	Não descrito	Até 30’	Não se aplica	As duas práticas de cuidados com os pés mais mencionadas nos vídeos foram “verificar os pés diariamente” (n = 34; 38,20%) e “usar sapatos e meias adequados” (n = 26; 29,21%). Apenas 14,6% dos vídeos continham informações úteis, com exceção de informações parcialmente úteis. Esses 14,6% foram fornecidos por podólogos ou clínicas especializadas em pés. Somente 10,1% do total de vídeos eram de usuários em geral.
E21^(25)^	“Guidance Motion Picture” (GMP)	Não descrito	O GI (orientação sobre o filme + folheto informativo), GC positivo 1 (orientação sobre o filme) e o GC positivo 2 (folheto informativo).	O conhecimento foi significativo para o GI (p = 0,005), para o GC positivo 1 (p = 0,011) e para o CG positivo 2 (p = 0,097). A análise da atitude foi significativa para todos os grupos (p = 0,005). A análise da ação foi significativa para o GI (p = 0,005) e para o grupo controle positivo 1 (p = 0,005), o GC positivo 2 (p = 0,011). Há diferenças na eficácia entre a combinação de vídeo educativo + folheto informativo e o vídeo educativo isolado, em relação ao aumento do conhecimento (p = 0,047), da atitude (0,022) e da ação (p = 0,037).
E22^(27)^	Não descrito	~10’	Os participantes foram aleatoriamente divididos em dois grupos de igual tamanho: G1 assistiu à versão do vídeo educativo com foco nos ganhos. G2 assistiu à versão com foco nas perdas. Cada participante completou 4 sessões de teste: antes de assistir ao vídeo (dados demográficos, atitude, conhecimento e comportamento), imediatamente após assistir ao vídeo (atitude e conhecimento) e em acompanhamentos de 3 e 6 meses (atitude, conhecimento e comportamento).	Vídeos com mensagens de prevenção com foco nos ganhos são mais eficazes do que mensagens com foco nas perdas para promover comportamentos que mantêm a saúde em pessoas com diabetes.
E23^(28)^	Não descrito	Não descrito	Os participantes foram divididos em 3 grupos. O GC recebeu o material educativo escrito atualmente fornecido pelo hospital a pacientes com diabetes. O GI recebeu educação em vídeo que abrangia o mesmo conteúdo do material escrito. Um terceiro grupo recebeu educação por escrito e pelo vídeo. O vídeo foi assistido de 1 a 3 vezes enquanto a pessoa estava no hospital	A maioria dos pacientes apresentou mudança significativa nos testes pós-educacionais usando a ferramenta de educação em vídeo (GI). Um estudo de acompanhamento é necessário para fornecer mais evidências das preferências dos pacientes em relação à educação (apenas vídeo ou vídeo + folheto escrito).
E24^(33)^	Não descrito	28’	Para testar a utilidade do programa, foi elaborado um questionário. Os pacientes foram solicitados a responder às mesmas oito perguntas (sim/não) antes e depois de assistirem ao vídeo.	Após a visualização do vídeo, aumentou de 69% para 91% a quantidade de pessoas que souberam responder as perguntas sobre cuidados com os pés. Após 6 meses, a maioria enfatizou que havia seguido pelo menos algumas das recomendações de autocuidado com os pés, que não haviam realizado anteriormente.

Legenda: DM: diabetes mellitus; DM2: diabetes mellitus tipo 2; IVC: Índice de Validade de Conteúdo; CVC: Coeficiente de Validade de Conteúdo; CVCt: Coeficiente de Validade de Conteúdo total; GC: grupo controle; GI: grupo intervenção; DP: desvio padrão; GMP: Guidance Montion Picture; M-DFCE: Mobile Diabetic Foot Care Education.

Fonte: Autores (2025).

Em relação ao conteúdo abordado nos vídeos, os estudos apresentaram orientações gerais e sobre diabetes^(4,15,16,18,19,25,26,30,31,34,35,36)^ (12;50,0%), autocuidado com os pés, de forma geral, sem especificar as orientações^(4,16,17,18,20,23,25,26,27,29,30,31,37)^ (13;54,1%). Quanto às ações de autocuidado descritas no vídeo, predominaram cuidado com as unhas^(15,16,17,19,22,24,26,30,32,35,36)^ (11;45,8%) seguido de inspeção nos pés^(15,16,17,22,24,26,30,34,35,36)^, lavagem dos pés^(15,16,17,22,24,26,30,32,35,36)^, uso adequado de calçados^(15,16,17,19,22,24,26,30,35,36)^ e uso de meias^(15,16,17,22,24,26,30,32,35,36)^ (10;41,6% cada). O conteúdo menos abordado nos vídeos foi orientação de exercícios para os pés^(16,26)^ (2;8,3%). Três estudos^(21,28,33)^ (12,5%) não citaram o conteúdo do vídeo.

A Figura 2 apresenta a síntese dos conteúdos abordados nos vídeos sobre autocuidado com os pés nos estudos da revisão de escopo.

**Figura 2:** Síntese dos conteúdos abordados nos vídeos sobre autocuidado com os pés nos estudos da revisão de escopo – Fortaleza, CE, Brasil, 2025.

**Fonte:** Autores (2025)

## DISCUSSÃO

Os achados dessa revisão de escopo evidenciam a crescente utilização de vídeos educacionais como estratégia de apoio ao autocuidado de pessoas com diabetes, especialmente no cuidado com os pés, aspecto essencial para a prevenção de úlceras e amputações^(38,39)^. Esses recursos apresentam potencial para ampliar o acesso à informação em saúde, favorecer o protagonismo do paciente e fortalecer ações educativas em diferentes contextos assistenciais.

Apesar desse potencial, os estudos incluídos identificaram fragilidades relacionadas à qualidade das informações disponibilizadas. Observou-se elevada heterogeneidade, ausência de validação clínica e de diretrizes padronizadas, quanto ao conteúdo, duração, linguagem, estratégias pedagógicas e recursos audiovisuais utilizados, além de diferenças nos critérios empregados para avaliação dos materiais. Estudos que analisaram vídeos disponíveis em plataformas abertas, como o YouTube, demonstraram maior variabilidade na qualidade científica e menor alinhamento às recomendações baseadas em evidências, quando comparados a materiais produzidos por instituições de saúde ou grupos acadêmicos^(40,41)^.

Além disso, muitos vídeos apresentavam informações incompletas ou superficiais sobre medidas preventivas fundamentais para o autocuidado dos pés, incluindo inspeção diária, higiene adequada, avaliação de calçados e identificação precoce de lesões. Observou-se que a maioria dos materiais não contemplava integralmente as recomendações da *International Working Group on the Diabetic Foot* ^(1)^, limitando o potencial educativo e a aplicabilidade clínica dessas tecnologias.

Os estudos também evidenciaram diferenças importantes quanto aos processos de desenvolvimento e validação dos vídeos. Materiais submetidos à avaliação de especialistas e testes com usuários apresentam maior clareza, organização e adequação das informações, favorecendo a compreensão e a aplicabilidade das orientações educativas^(30,42,43)^. Ademais, observou-se que grande parte dos vídeos disponíveis em plataformas digitais não passa por qualquer processo de validação, o que reforça a urgência de incorporar etapas de avaliação clínica como fase obrigatória no desenvolvimento de materiais educacionais.

Nesse contexto, destaca-se a necessidade de protocolos padronizados para a produção de vídeos educativos em saúde. Embora existam recomendações gerais sobre educação em saúde e comunicação digital, os estudos apontam escassez de modelos sistematizados que orientem aspectos como elaboração de roteiros, definição de linguagem, uso de recursos audiovisuais e adaptação do conteúdo às necessidades do público-alvo^(44,45)^. Essa ausência de padronização dificulta a comparação entre estudos, compromete a reprodutibilidade das intervenções e limita a consolidação de evidências sobre a efetividade dos vídeos educacionais.

Outro aspecto relevante refere-se ao impacto da desigualdade no acesso às tecnologias educacionais. Embora os vídeos possam ampliar o alcance das ações de educação em saúde, fatores como baixo nível socioeconômico, limitação do acesso à internet e dificuldades de alfabetização digital podem restringir sua utilização por parte de populações mais vulneráveis^(46,47)^. Dessa forma, a incorporação de tecnologias digitais na educação em saúde deve ser acompanhada por políticas públicas voltadas à inclusão e à ampliação do acesso às ferramentas tecnológicas.

No contexto brasileiro, observa-se avanço na incorporação da Saúde Digital como eixo estruturante do Sistema Único de Saúde (SUS), especialmente por meio de iniciativas como a Política Nacional de Saúde Digital e o Conecte SUS, que estimulam o desenvolvimento e a utilização de recursos tecnológicos aplicados ao cuidado em saúde^(48,49)^. Entretanto, os achados desta revisão demonstram que a expansão dessas ferramentas deve ocorrer associada ao fortalecimento de mecanismos de avaliação da qualidade das informações disponibilizadas e pormenorizadas.

Nesse sentido, estudo de Smith et al.^(26)^, ao avaliar vídeos sobre autocuidado com os pés em pessoas com diabetes disponíveis no YouTube, identificou que vídeos mais longos foram significativamente classificados como úteis em comparação aos considerados enganosos ou pouco úteis. Esse resultado sugere que materiais mais detalhados podem favorecer maior abrangência das orientações educativas, embora a duração, isoladamente, não seja suficiente para garantir qualidade científica.

Além da qualidade técnica, os estudos reforçam a importância do fortalecimento da capacidade crítica dos usuários frente às informações em saúde disseminadas no ambiente tecnológico. A ampla circulação de conteúdo sem curadoria científica pode dificultar a identificação de fontes confiáveis, tornando essencial o desenvolvimento de estratégias educativas que promovam alfabetização digital e uso crítico das tecnologias em saúde^(20,47,49)^.

Apesar das limitações identificadas, os vídeos educativos permanecem como ferramentas promissoras para apoiar o autocuidado com os pés em pessoas com diabetes, especialmente quando utilizados como complemento às orientações presenciais realizadas pelos profissionais de saúde. Seu impacto positivo depende diretamente da qualidade do conteúdo, validação clínica, utilização de estratégias pedagógicas adequadas e ampliação do acesso às tecnologias.

Os achados evidenciam a necessidade de desenvolver vídeos educativos de orientação ao autocuidado com os pés em consonância com as diretrizes atuais, adaptado culturalmente, capazes de atender às diferentes necessidades e contextos socioculturais das pessoas com diabetes. Além disso, ressaltam a importância da ampliação do acesso às tecnologias digitais como estratégia para potencializar as ações de educação em saúde, favorecer o engajamento no autocuidado e fortalecer a autonomia de pessoas com diabetes na prevenção de complicações relacionadas aos pés.

As principais limitações desta revisão de escopo incluem a heterogeneidade metodológica dos estudos incluídos, que dificultou a comparação direta entre os resultados, especialmente no que se refere aos diferentes formatos, durações e critérios de avaliação dos vídeos educacionais. Além disso, muitos materiais analisados não apresentavam informações suficientes sobre processos de validação ou base teórica utilizada, restringindo a avaliação da qualidade e credibilidade dos conteúdos. A predominância de estudos publicados em determinados idiomas pode ter levado à sub-representação de produções oriundas de países de baixa e média renda, onde a desigualdade digital é mais acentuada.

Por fim, a busca em bases de dados pode não ter capturado vídeos disseminados exclusivamente em plataformas digitais não indexadas, resultando em possível perda de materiais relevantes, o que sugere que os achados devem ser interpretados com cautela.

## CONCLUSÃO

Os vídeos educacionais disponíveis e descritos na literatura representam um recurso potencialmente valioso para o fortalecimento do autocuidado com os pés em pessoas com diabetes. Apresentam potencial para fortalecer o autocuidado e ampliar o acesso à educação em saúde. Entretanto, sua efetividade depende de maior padronização, curadoria profissional e processos de validação.

Investir em materiais audiovisuais baseados em evidências e alinhados a diretrizes clínicas é fundamental para aprimorar a qualidade, segurança e aplicabilidade dessas tecnologias na prática clínica e no cuidado às pessoas com diabetes.

## DISPONIBILIDADE DE DADOS

Todo o conjunto de dados que dá suporte aos resultados deste estudo foi publicado no próprio artigo.

# Translation: Tecnología educativa en video para el autocuidado de los pies en personas con diabetes: revisión de alcance

**DOI:** 10.1590/1980-220X-REEUSP-2026-0169es

**Authors:** Amelina de Brito Belchior, Florência Gamileira Nascimento, Victoria Maria Silva Leitão, Thiago Martins de Sousa, Lourival Veras de Oliveira, Cecília Lima de Souza, Sherida Karanini Paz de Oliveira

**Affiliations:** 1 Universidade Estadual do Ceará, Programa de Pós-Graduação em Cuidados Clínicos em Enfermagem e Saúde, Fortaleza, CE, Brasil.; 2 Universidade Estadual do Ceará, Centro de Ciências da Saúde, Departamento de Enfermagem, Fortaleza, CE, Brasil

**Keywords:** Complicaciones de la diabetes, Pie diabético, Autocuidado, Película y Video Educativos, Tecnología Biomédica

## Abstract

**Objetivo:**

Identificar, en la literatura, estudios que aborden los videos educativos existentes sobre el autocuidado de los pies en personas con diabetes.

**Método:**

Se llevó a cabo una revisión de alcance de acuerdo con las directrices del JBI. La búsqueda se realizó en nueve bases de datos y repositorios, con criterios de inclusión definidos por el enfoque PCC (Participantes, Concepto y Contexto). La recolección de datos se llevó a cabo entre marzo y junio de 2025.

**Resultados:**

Se incluyeron 24 estudios distribuidos en diferentes países, predominando Brasil e Indonesia (6; 25,0 % cada uno). Ocho estudios (33,3 %) describieron el nombre del video. En cuanto al contenido, se destacan acciones específicas de autocuidado de los pies, como el cuidado de las uñas (11; 45,8 %). Tres estudios no mencionaron el contenido del video.

**Conclusión:**

Los estudios disponibles que abordan los videos educativos descritos en la literatura representan un recurso potencialmente valioso para fortalecer el autocuidado de los pies en personas con diabetes. Tienen el potencial de fortalecer el autocuidado y ampliar el acceso a la educación en salud. Sin embargo, su efectividad depende de una mayor estandarización, curaduría profesional y procesos de validación.

## INTRODUCCIÓN

La diabetes mellitus (DM) es una de las enfermedades crónicas que desde hace mucho tiempo constituyen uno de los grandes problemas de salud pública y está asociada a diversas complicaciones que comprometen de manera significativa la calidad de vida de las personas. Entre estas complicaciones, se destaca la enfermedad del pie relacionada con la diabetes, la cual genera impactos económicos, sociales y psicológicos significativos para los sistemas de salud, el paciente y la familia^(1)^. Se estima que 131 millones de personas en el mundo presentan complicaciones en las extremidades inferiores asociadas a la diabetes, como la neuropatía periférica y la úlcera del pie relacionada con la diabetes, lo que corresponde al 1,8 % de la población mundial^(2)^. Esta cifra, en constante crecimiento, sumada a las dificultades en el manejo clínico, las limitaciones de los servicios de salud y las desigualdades sociales, contribuye a que las tasas de amputación sigan considerándose inaceptables^(3)^.

En este contexto, el autocuidado desempeña un papel esencial en la prevención y la detección temprana de complicaciones en los pies, siendo la educación estructurada, organizada y continua fundamental para evitar úlceras y amputaciones relacionadas con la diabetes^(1)^. Las medidas preventivas y las acciones educativas, incluso a través de intervenciones tecnológicas, resultan indispensables para reducir los riesgos para la salud y fortalecer el autocuidado de los pies en personas con DM^(4,5)^.

Las tecnologías han evolucionado rápidamente, lo que hace esencial que el sector de la salud se mantenga al día con estas innovaciones. Sin embargo, su incorporación efectiva a la práctica clínica exige el desarrollo de materiales de calidad y la generación de evidencia sistematizada sobre su aplicación^(6)^. Así, los materiales audiovisuales, al ser accesibles y de amplio alcance, pueden apoyar la prevención de complicaciones, fortalecer la participación del paciente y complementar las estrategias tradicionales de educación en salud. Al identificar y sistematizar estas iniciativas, se potencia la ampliación del uso de tecnologías digitales en el cuidado de la diabetes, promoviendo prácticas más eficaces, equitativas y sostenibles^(7)^.

En este sentido, el uso de videos educativos, como tecnología educativa en salud, es una posibilidad concreta de apoyo y aprendizaje que busca compartir mensajes y orientaciones de la manera más clara y objetiva posible, para que el espectador se sienta motivado y pueda poner en práctica de manera eficiente las orientaciones brindadas^(8)^. Esto puede beneficiar la vida de las personas con diabetes, facilitar el aprendizaje con instrucciones correctas para el autocuidado de los pies, mejorar y transformar la forma en que se cuidan a sí mismas, haciéndolas más motivadas y comprometidas con su condición de salud^(4)^.

Por lo tanto, resulta relevante desarrollar acciones de orientación sobre el autocuidado de los pies para personas con diabetes mediante tecnologías audiovisuales. Por lo tanto, es necesario identificar los videos educativos disponibles en la literatura científica, analizando sus características, potencialidades, contenidos abordados y las lagunas que aún existen.

De esta manera, este estudio tiene como objetivo mapear, en la literatura, los videos educativos existentes sobre el autocuidado de los pies en personas con diabetes.

## MÉTODO

### Tipo de Estudio

Se trata de una revisión de alcance realizada según las recomendaciones del *Joanna Briggs Institute* (JBI) y estructurada con base en la *lista de verificación Preferred Reporting Items for Systematic reviews and Meta-Analyses extension for Scoping Reviews* (PRISMA-ScR). El protocolo de investigación se registró en el *Open Science Framework* (OSF) con la identificación: https://doi.org/10.17605/OSF.IO/BGTHK.

Se desarrolló en nueve etapas: 1) Identificación del título y la pregunta de investigación; 2) Definición de los criterios de elegibilidad de acuerdo con la estrategia PCC; 3) Selección de fuentes de evidencia; 4) Definición de la estrategia de búsqueda; 5) Clasificación y selección de la evidencia; 6) Extracción de datos mediante un formulario específico; 7) Análisis y síntesis de los datos; 8) Presentación de los resultados; y 9) Resumen de la evidencia en relación con el propósito de la revisión, las conclusiones y las implicaciones de los hallazgos^(9)^.

### Etapas del Estudio

#### Primera Etapa: Identificación del Título y la Pregunta de Investigación

En la primera etapa, se utilizó la estrategia PCC^(9)^, tomando en cuenta: Población: personas con diabetes; Concepto: videos educativos; y Contexto: autocuidado de los pies. Así, se formuló la siguiente pregunta de investigación: “¿Qué estudios que abordan videos educativos sobre el autocuidado de los pies en personas con diabetes están disponibles en la literatura?”. La pregunta secundaria fue: ¿Cuáles son los contenidos abordados en los videos educativos para el autocuidado de los pies en personas con diabetes?

#### Segunda Etapa: Definición de los Criterios de Elegibilidad

La segunda etapa consistió en la definición de los criterios de inclusión y exclusión, de acuerdo con la estrategia PCC. Se incluyeron estudios con videos dirigidos a personas de cualquier edad y tipo de diabetes que abordaran el autocuidado de los pies, distribuidos en cualquier formato, tales como módulos en línea, aplicaciones, redes sociales o contenidos almacenados en dispositivos móviles, en todos los idiomas y sin restricción temporal. Se excluyeron los estudios con tecnologías destinadas exclusivamente a profesionales de la salud, aquellos que no especificaban el tipo de tecnología utilizada, así como comentarios, cartas al editor, resúmenes, pósteres de congresos y estudios de revisión, debido a la limitación de la información disponible. Sin embargo, se analizaron sus referencias para identificar y seleccionar estudios que pudieran ser relevantes para su inclusión.

#### Tercera Etapa: Selección de Fuentes de Evidencia

La tercera etapa, la selección de fuentes de evidencia, se llevó a cabo en las siguientes bases de datos y repositorios de búsqueda del área de la salud: *Medical Literature Analysis and Retrieval System Online* (MEDLINE) a través de PubMed, Literatura Latinoamericana y del Caribe en Ciencias de la Salud (LILACS) a través de la Biblioteca Virtual en Salud (BVS), *Web of Science, Scopus, Cumulative Index to Nursing and Allied Health Literature* (CINAHL) y Embase. La literatura gris se recuperó de cuatro fuentes: *Google Scholar*, Biblioteca Digital Brasileña de Tesis y Disertaciones (BDTD), Catálogo de Tesis y Disertaciones (CTD) de la Coordinación para el Perfeccionamiento del Personal de Nivel Superior (CAPES) y *ProQuest Dissertations and Theses* (PQDT).

#### Cuarta Etapa: Definición de la Estrategia de Búsqueda

Para definir la estrategia de búsqueda, se utilizaron descriptores, palabras clave y sinónimos de acuerdo con cada base de datos, mediante una búsqueda en los tesauros de salud y diccionarios reconocidos en el ámbito de la salud, como *Medical Subject Headings* (MESH), Descriptores en Ciencias de la Salud (DECS) y EMTREE. Cabe destacar que se aplicaron los filtros de tesis y disertaciones en la plataforma ProQuest.Para la formulación de una estrategia de búsqueda sensible, se utilizó la estrategia ECUS (extracción, conversión, combinación, construcción y uso)^(10)^, empleando los operadores booleanos OR y AND (Cuadro 1).

**Cuadro 1:** Estrategia de búsqueda para la elaboración de la revisión de alcance – Fortaleza, CE, Brasil, 2025^(10)^.

Objetivo/problema	Recopilar videos educativos sobre el autocuidado de los pies para personas con diabetes			
	P	C	C	
Extracción	Personas con diabetes mellitus	Videos educacionales	Autocuidado de los pies	
Conversión	Diabetes mellitus	video educacional	Selfcare (autocuidado)	diabetic foot (pie diabético)
Combinación	Diabetes mellitus, Diabetic Neuropathies, diabetes, diabetic, diabetic patient	Webcasts, video, educational video, infographics, video technology, streaming video, video animation	Selfcare, self-care, self-management;	diabetic foot, Diabetic foot, Diabetic Feet, Ulcer Diabetic foot, diabetic foot wound, diabetic foot ulcer, diabetic foot sore
Construcción	(“diabetes mellitus” OR “Diabetic Neuropathies” OR diabetes OR diabetic OR “diabetic patient”)	(Webcasts, OR vídeo OR “educational video” OR infographics OR “video technology” OR “streaming video” OR “video animation”)	(Selfcare OR self-care OR self-management OR selfmanagement) AND (“diabetic foot” OR Diabetic foot” OR “Diabetic Feet” OR “Ulcer Diabetic foot” OR “diabetic foot wound” OR “diabetic foot ulcer” OR” diabetic foot sore”)	
#Uso 1	(“diabetes mellitus” OR “Diabetic Neuropathies” OR diabetes OR diabetic OR “diabetic patient”) AND (Webcasts, OR vídeo OR “educational video” OR infographics OR “vídeo technology” OR “streaming video” OR “video animation”) AND (Selfcare OR self-care OR self-management OR selfmanagement) AND (“diabetic foot” OR Diabetic foot” OR “Diabetic Feet” OR “Ulcer Diabetic foot” OR “diabetic foot wound” OR “diabetic foot ulcer” OR “diabetic foot sore”).			
#Uso 2	(“diabetes mellitus OR “neuropatias diabéticas” OR diabetes OR diabético) AND (Webcast OR vídeos OR “vídeos educacionais” OR Infograficos OR “animação em vídeo” OR “tecnologia em vídeo”) AND (autocuidado OR autogerenciamento) AND (“pé diabético” OR “pés diabéticos” OR “úlcera do pé diabético”).			

#### Quinta Etapa: Cribado y Selección de la Evidencia

La selección de los estudios se llevó a cabo mediante el *software Rayyan*, que permite un cribado ciego e independiente entre los revisores, mediante el análisis de títulos, resúmenes y la lectura completa de los estudios encontrados en la literatura, eliminando los duplicados.

#### Sexta Etapa: Extracción de Datos Mediante un Formulario Específico

En la sexta etapa, dedicada a la extracción de datos mediante un formulario específico, dos revisores participaron de manera independiente en la evaluación de la elegibilidad de los estudios, utilizando el software *Endnote web* (http://www.myendnoteweb.com/EndNoteWeb.html) y hojas de cálculo de Excel para la gestión de los estudios. Inicialmente, los evaluadores realizaron una selección preliminar a partir de la lectura de los títulos y resúmenes. Tras una reunión de consenso, se confirmó la selección de los artículos, justificándose la exclusión de acuerdo con los criterios establecidos. Se leyeron los estudios en su totalidad y, posteriormente, se analizaron las referencias para incluir nuevos estudios. Esta etapa se llevó a cabo entre marzo y junio de 2025.

La extracción y el manejo de los datos se realizaron mediante un mapeo que contenía información de caracterización, como autoría, año, país, título, objetivo, tipo de estudio, idioma, nivel de evidencia basado en la clasificación jerárquica de Melnyk y Fineout-Overholt^(11)^ y características de los videos.

#### Séptima Etapa: Análisis y Síntesis de los Datos

En la séptima etapa, de análisis y síntesis de los datos, se evaluó la concordancia entre los revisores mediante el análisis del coeficiente de *Kappa de Cohen*, adoptando los siguientes parámetros: 0–0,20, nula; 0,21–0,39, mínima; 0,40–0,59, débil; 0,60–0,79, moderado; 0,80–0,90, fuerte; por encima de 90, casi perfecto^(12)^.

En la extracción de datos, se elaboró un resumen crítico que sintetizaba toda esta información. Es importante destacar además que el nivel de evidencia según esta referencia se determina de acuerdo con las características metodológicas de los estudios; cuanto mayor es el nivel de evidencia, mejor representación tienen los estudios con mayor grado de confiabilidad para la toma de decisiones. Además, se consideraron también características de la propia producción textual de los estudios, como el nombre del video creado, la caracterización de la tecnología, la duración, el público objetivo y el lenguaje utilizado.

Los resultados se presentaron mediante materiales gráficos como cuadros, tablas y figuras, y se analizaron a la luz de la literatura pertinente actualizada.

La octava etapa, presentación de los resultados, y la novena, resumen de la evidencia en relación con el propósito de la revisión, las conclusiones y las implicaciones de los hallazgos, se exponen a continuación.

### Aspectos Éticos

Para la realización de este estudio, se respetaron todos los preceptos éticos. Todos los autores de los artículos analizados fueron citados adecuadamente, de conformidad con la Ley de Derechos de Autor n.º 9.610, del 19 de febrero de 1998^(13)^. Los datos y la información de las investigaciones se presentaron de manera fidedigna.

Durante la elaboración de este artículo, los autores no utilizaron herramientas de generación de texto ni de Inteligencia Artificial (IA).

## RESULTADOS

La búsqueda identificó 1.906 estudios potencialmente relevantes, de los cuales se eliminaron 653 por duplicación. Los 1.253 estudios restantes se seleccionaron por título y resumen. De ese total, se incluyeron 50 estudios para el análisis del texto completo por parte de dos revisores independientes. Posteriormente, tras la lectura completa, se excluyeron 31 estudios por no responder a la pregunta de investigación, quedando 19 estudios.

A partir de la consulta de las listas de referencias, se identificaron cinco estudios para componer la muestra, lo que dio como resultado 24 estudios incluidos en esta revisión de alcance. La Figura 1 muestra el proceso de selección.

**Figura 1:** Diagrama de flujo PRISMA-ScR para la selección de los estudios de la revisión de alcance – Fortaleza, CE, Brasil, 2025.

**Fuente:** datos de la investigación (2025), adaptado de Page et al.^(14)^.

Los estudios incluidos provienen de diferentes países, entre los que predominan Brasil^(4,15–19)^ e Indonesia^(20–25)^ (6; 25,0 % cada uno), seguidos de Estados Unidos^(26–28)^, (3; 12,5 %), Turquía (29, 30) (2; 8,3 %), Portugal^(29)^, Túnez^(30)^, India^(31)^, Marruecos^(32)^ y los Países Bajos^(33)^ (1; 4,1 % cada uno). Se realizaron dos estudios en dos países: Arabia Saudita y el Reino Unido^(34)^ y Arabia Saudita y Canadá^(35)^ (1; 4,2 % cada uno).

El estudio más antiguo se publicó en 1987^(33)^ (1; 4,1 %), con mayor prevalencia en los años 2022^(17–19,34,36)^ y 2023^(15,16,20,29,30)^ (5; 20,8 cada uno). Otros tuvieron una menor frecuencia de publicación, en 2011^(27,28)^, 2019^(24,26)^ (2; 8,3 % cada uno), 1987^(33)^, 2014^(25)^, 2015^(35)^, 2017^(32)^, 2021^(21)^, 2024^(4)^ (1; 4,1 % cada uno).

El tipo de publicación más frecuente fue el artículo^(4,17,19–37)^ (21;87,5 %), seguido de la tesis^(15,16,18)^ (3;12,5 %), publicados en inglés^(24,26–29,31–37)^ (12; 50,0 %), portugués^(4,15–19)^ (6; 25,0 %), indonesio^(20–23,25)^ (5; 20,8 %) y francés^(30)^ (1; 4,1 %).

En cuanto al método, destacan los estudios cuasi-experimentales^(20–25,28,30)^ (8; 33,3 %), seguidos de los metodológicos^(15–19)^ y los ensayos clínicos aleatorizados^(27,29,31,32,37)^ (5; 20,8 % cada uno). Cuatro artículos^(26,34–36)^(16,6 %) evaluaron los videos sobre el autocuidado de los pies como fuente de información disponible en la plataforma *You Tube*, y un artículo^(29)^ (4,1 %) trata sobre el protocolo de un ensayo clínico aleatorizado.

Los estudios encontrados presentaron con mayor frecuencia un nivel de evidencia II^(27,29,31,32,37)^ (5; 20,8 %), III^(20–26,28,30)^ (9; 37,5 %) y VI^(4,15–19,33–36)^ (10; 41,6 %).

La Cuadro 2 presenta la caracterización de los estudios incluidos en la revisión, en cuanto a autoría, año de publicación, país, título, objetivos, muestra, idioma, método y nivel de evidencia.

**Cuadro 2:** Caracterización de los estudios seleccionados para la revisión de alcance – Fortaleza, CE, Brasil, 2025.

ID	Autor (año)/país	Título	Objetivo	Muestra	Idioma	Método/nivel de evidencia
E1	Andrade et al. (2024)^(4)^. Brasil	Percepção dos usuários com Diabetes sobre o Autocuidado com os Pés: Uma análise qualitativa	Evaluar la percepción sobre el uso de videos educativos para el autocuidado de los pies en personas con DM2 en la atención primaria en el estado de Amazonas.	20 personas con DM2.	Portugués	Estudio cualitativo (Artículo)/VI
E2	Ferreira et al. (2023)^(29)^. Portugal	Educational Interventions on Diabetic Foot Self-Care: A Study Protocol for a Pragmatic Randomized Controlled Trial	Analizar el impacto de diferentes estrategias educativas: un video instructivo	54 personas con DM y úlceras en los pies, 18 en cada grupo	Inglés	Ensayo clínico aleatorizado (Artículo)/II
E3	Octaviana y Hati (2023)^(20)^. Indonesia	Analisis Pengaruh Edukasi Melalui Media Video terhadap Tingkat Pengetahuan Pasien Diabetes Melitus Tentang Perawatan Kaki di Puskesmas	Analizar la eficacia de la educación sobre el cuidado de los pies mediante videos en el nivel de conocimiento de los pacientes con diabetes mellitus.	60 personas con DM	Indonesio	Estudio cuasi-experimental
E4	Oliveira (2023)^(15)^. Brasil	Construção e validação de vídeo educativo para prevenção do pé diabético centrado no apoio social	Crear y validar un video educativo para la prevención del pie diabético centrado en el apoyo social de las personas con DM.	26 especialistas (profesionales de la salud)	Portugués	(Artículo)/III
E5	Yazidi et al. (2023)^(30)^. Túnez	Place des supports vidéo disponibles en ligne dans l’éducation thérapeutique des diabétiques: étude évaluative chez une population de diabétiques à haut risque podologique	Evaluar el impacto de un video educativo en línea en la mejora de los conocimientos de los pacientes diabéticos con alto riesgo de problemas en los pies en relación con la prevención de lesiones en los pies.	40 personas con diabetes con alto riesgo de sufrir problemas en los pies.	Francés	Estudio metodológico (Tesis)/VI
E6	Siqueira (2023)^(16)^. Brasil	Vídeo Educativo: atividades de autocuidado para prevenção de complicações nos pés de pessoas idosas com diabetes	Crear un video educativo sobre las actividades de autocuidado para la prevención de complicaciones en los pies de las personas mayores con diabetes.	9 especialistas (enfermeros)	Portugués	Estudio cuasi-experimental (Artículo)/III
E7	Almaqhawi et al. (2023)^(34)^. Arabia Saudita y Reino Unido	Evaluation of quality of diabetic foot examination on YouTube	Evaluar la calidad educativa y la confiabilidad de los videos disponibles públicamente en YouTube sobre exámenes de los pies en personas con diabetes, con el fin de verificar si YouTube es o no una fuente confiable de información de salud para los pacientes.	100 videos de YouTube	Inglés	Estudio metodológico (Tesis)/VI
E8	Doğan y Pehlivan (2022)^(36)^. Turquía	Diabetic foot care training and the presence of nurses in Turkish YouTube videos	Analizar el contenido de videos turcos de YouTube como fuente de información sobre capacitación en el cuidado de los pies de pacientes diabéticos y la presencia de enfermeros en dichos videos.	87 videos de YouTube	Inglés	Estudio descriptivo (Artículo)/VI
E9	Menezes et al. (2022)^(17)^. Brasil	Produção e validação do curta-metragem Pés que te quero®: tecnologia educacional para pessoas com diabetes	Describir la producción y validación de tecnología educativa en formato de cortometraje para la prevención de úlceras del pie diabético.	31 especialistas en enfermería, 6 en comunicación y 15 personas con DM2	Portugués	Estudio descriptivo (Artículo)/VI
E10	Nascimento (2022)^(18)^. Brasil	Construção e validação do vídeo cordel “ô pé arretado” como tecnologia educativa para o pé diabético	Crear y validar una obra de literatura popular en cordel con presentación en video como tecnología educativa para la prevención del pie diabético.	8 estudiantes de enfermería y 6 especialistas para la validación del contenido y la apariencia, y cuatro personas con DM para evaluar la usabilidad	Portugués	Estudio metodológico
E11	Meneses et al. (2022)^(19)^. Brasil	Desenvolvimento e validação de vídeo educativo sobre cuidados podiátricos para prevenção de úlceras em idosos com diabetes	Desarrollar y validar un video educativo sobre cuidados podológicos para la prevención del pie diabético en personas mayores.	10 especialistas.	Portugués	(Artículo)/VI
E12	Wicahyani et al. (2021)^(21)^. Indonesia	Aplikasi Edukasi Berbasis Video Untuk Meningkatkan Dukungan Keluarga Mencegah Komplikasi Kaki Diabetes	Investigar la eficacia de la educación basada en videos para aumentar el apoyo familiar en la prevención de complicaciones en los pies de pacientes con diabetes mellitus tipo 2.	80 personas con diabetes y sus familiares.	Indonesio	Estudio metodológico (Tesis)/VI
E13	Hamzah et al. (2020)^(22)^. Indonesia	The effectiveness of foot care education video in Makassar language to increase patients’ knowledge related foot care in community	Determinar la eficacia de un video educativo sobre el cuidado de los pies, en lengua macasar, para mejorar los conocimientos de los pacientes con DM sobre el tema.	30 personas con diabetes sin úlceras en los pies	Indonesio	Estudio metodológico (Artículo)/VI
E14	Dincer y Bahçecik (2020)^(37)^. Turquía	The effect of a mobile application on the foot care of individuals with type 2 diabetes: A randomised controlled study	Desarrollar una aplicación móvil educativa sobre el cuidado de los pies en personas con diabetes (M-DFCE), con animaciones de apoyo, para que la utilicen personas con diabetes tipo 2, e identificar sus efectos sobre los conocimientos, la autoeficacia y el comportamiento en el cuidado de los pies.	130 personas con diabetes (65 en el grupo experimental y 65 en el grupo de control).	Inglés	Estudio cuasi-experimental (Artículo)/III
E15	Nurjanna et al. (2020)^(23)^. Indonesia	Perbandingan pengetahuan self efficacy perawatan kaki pada pasien Diabetes Melitus tipe II setelah menggunakan video edukasi di puskesmas paccerakkang kota makassar	Comparar los conocimientos sobre la autoconfianza en el cuidado de los pies en pacientes con diabetes mellitus tipo 2 tras el uso de videos educativos en el Centro de Salud Comunitario de Paccerakkang, en la ciudad de Makassar.	20 pacientes con DM	Indonesio	Estudio cuasi-experimental (Artículo)/III
E16	Abrar et al. (2020)^(24)^. Indonesia	Development and evaluation educational videos of diabetic foot care in traditional languages to enhance knowledge of patients diagnosed with diabetes and risk for diabetic foot ulcers	Desarrollar un video educativo sobre el cuidado de los pies en personas con diabetes en las lenguas tradicionales (buginese y macasares) y evaluar el efecto de estos videos en los conocimientos del paciente	7 enfermeros especialistas para el desarrollo del contenido del video, tres para la validez del contenido y 20 personas de cada grupo étnico con DM con riesgo de úlcera en los pies	Inglés	Ensayo clínico aleatorizado (Artículo)/II
E17	Smith et al. (2019)^(26)^. EUA	Analysis of YouTube as a Source of Information for Diabetic Foot Care		87 videos de Youtube	Inglés	Estudio descriptivo (Artículo)/VI
E18	Rahaman et al. (2018)^(31)^. India	Effectiveness of a Patient Education Module on Diabetic Foot Care in Outpatient Setting: An Open-label Randomized Controlled Study	Alertar a los médicos sobre la calidad de la información sobre el cuidado de los pies en personas con diabetes disponible en YouTube y determinar si YouTube es una fuente útil de información para pacientes con problemas en los pies debido a la diabetes.	127 (participaron en todas las etapas: 101 pacientes con DM1 y DM2 GI: 63 (participaron en todas las etapas: 51). GC: 64 (participaron en todas las etapas: 50)	Inglés	Ensayo clínico aleatorizado (Artículo)/II
E19	Adarmouch et al. (2017)^(32)^. Marruecos	Short-term effectiveness of a culturally tailored educational intervention on foot self-care among type 2 diabetes patients in Morocco	Desarrollar un módulo educativo para pacientes sobre el cuidado de los pies en la diabetes y evaluar su eficacia en el conocimiento y la práctica del cuidado de los pies en pacientes con diabetes.	Se reclutaron 199 personas con DM que participaron en la primera evaluación y 133 completaron la segunda evaluación	Inglés	Ensayo clínico aleatorizado (Artículo)/II
E20	Abedin et al. (2015)^(35)^. Aaábia Saudita y Canadá	YouTube as a source of useful information on diabetes foot care on diabetes foot care	Evaluar la eficacia de una intervención educativa para el autocuidado, adaptada culturalmente a las prácticas de autogestión del cuidado de los pies en pacientes con diabetes tipo 2, e identificar los factores asociados a la variación de dichas prácticas.	89 videos de YouTube	Inglés	Investigación documental (Artículo)/VI
E21	Setyorini et al. (2014)^(25)^. Indonesia	Edukasi perawatan kaki melalui media guidance motion picture dan leaflet terhadap perilaku perawatan kaki non ulkus pada penderita Diabetes Melitus Tipe 2 di Puskesmas Loceret	Analizar el uso de YouTube como fuente eficaz de información sobre el cuidado de los pies en personas con diabetes.	30 pacientes con DM2	Indonesio	Estudio cuasi-experimental
E22	Grady et al. (2011)^(27)^ EUA	Using message framing to achieve long-term behavioral changes in persons with diabetes	Evaluar la eficacia de la educación sobre el cuidado de los pies mediante recursos audiovisuales y folletos sobre el cuidado de los pies sin úlceras en pacientes con DM2 en el Centro de Salud Comunitario de Loceret	155 personas con diabetes desde hace 5 años o más y sin antecedentes de problemas en los pies.	Inglés	(Artículo)/III
E23	Gravely et al. (2011)^(28)^. EUA	Comparison of three types of diabetic foot ulcer education plans to determine patient recall of education	Examinar el impacto de la forma en que se presenta la información en un programa educativo sobre el cuidado adecuado de los pies y su importancia en la prevención de complicaciones de la diabetes, considerando los cambios a largo plazo en el conocimiento, las actitudes y el comportamiento con respecto al cuidado de los pies.	30 personas con DM	Inglés	Ensayo clínico aleatorizado (Artículo)/II
E24	Bakker (1987)^(33)^. Holanda	Video Education on Foot-Care	Comparar el material educativo escrito que se utiliza actualmente con un video educativo y una combinación de material escrito y video para determinar el recuerdo que tiene el paciente de la educación recibida.	40 pacientes con DM	Inglés	Estudio cuasi-experimental (Artículo)/III

Abreviaturas: DM: diabetes mellitus; DM2: diabetes mellitus tipo 2; DM1: diabetes mellitus tipo 1; GC: grupo de control; GI: grupo de intervención; M-DFCE: Educación móvil sobre el cuidado de los pies en personas con diabetes.

Fuente: Autores (2025).

Se observó que en ocho estudios (33,3 %) se describían los nombres de los videos: “*Verdadeiro ou Falso? e Diabetes e Cuidados diários com os pés” ^(4)^, “Pés de quem Cuido” ^(15)^, “Autocuidado com os pés de idosos com diabetes” ^(16)^, “Pés que te quero” ®^(17)^, “Ô pé arretado” ^(18)^, “No rastro da prevenção: Uma narrativa sobre o diabetes e os cuidados com os pés na pessoa idosa*”^(19)^ (“¿Verdadero o falso? y Diabetes y cuidados diarios de los pies”^(4)^, “Los pies de quienes cuido”^(15)^, “Autocuidado de los pies en personas mayores con diabetes”^(16)^, “Pies que te quiero”®^(17)^, “¡Qué pie tan genial!”^(18)^, “Tras la pista de la prevención: una narrativa sobre la diabetes y el cuidado de los pies en las personas mayores”)^(19)^, “*Guidance Motion Picture*” (GMP)^(25)^ y el *Mobile Diabetic Foot Care Education* (M-DFCE)^(37)^, que contiene videos animados sobre pautas de autocuidado de los pies.

Entre los videos cuya duración se informó^(15–19,21,22,24,26,27, 29–31,33–37)^ (18; 75,0 %), la duración varió de 2 minutos y 51 segundos^(18)^ hasta 28^(33)^ y 30 minutos (37). Predominaron los videos con una duración de entre 3 y 10 minutos^(16,18,19,22,24,26,27,29–31,37)^ (11; 45,8 %). Cabe destacar que de los cuatro estudios (16,6 %) que evaluaron los videos de *You Tube* ^(23,31,34,36)^, dos (8,3 %) presentaron la duración promedio de los videos, 6,8 ± 6,6’^(34)^ y 8,08 ± 13,27’^(36)^, y dos (8,3 %) indicaron la duración máxima de los videos disponibles en la plataforma, hasta 10’^(26)^ y hasta 30’^(35)^.

En cuanto a la intervención llevada a cabo en los estudios, prevaleció el diseño de preprueba y posprueba ^(20–24,28,30,32,33)^ (9; 37,5 %) con la aplicación de cuestionarios para la evaluación antes y después de la exposición al video educativo. Otros estudios compararon el uso de videos con materiales impresos, orientaciones tradicionales o tratamiento estándar^(25,29,31,37)^ (4;16,6 %), mientras que algunos se centraron en la validación del contenido y la apariencia de los videos por parte de especialistas^(15,16,19)^ (3; 12,5 %) y por parte de expertos y el público objetivo^(17,18)^ (2; 8,3 %). También se identificaron estudios de análisis de la calidad y la utilidad de los videos disponibles en línea^(26,34–36)^ (4; 16,6 %). Un estudio^(4)^ (4,1 %) aplicó un cuestionario después de que el paciente viera dos videos educativos, con el objetivo de comprender la percepción de los usuarios sobre el cuidado de los pies antes y después de ver los videos. Otro estudio^(27)^ (4,1 %) presentó dos videos a grupos diferentes: uno con mensajes de prevención enfocados en las ganancias y otro en las pérdidas, con el fin de promover comportamientos que mantengan la salud en personas con diabetes. Un artículo^(29)^ (4,1 %) presentó un protocolo de estudio sobre el impacto de dos estrategias educativas diferentes, sin proporcionar información sobre los resultados.

Los principales resultados reportados abarcan un aumento del conocimiento sobre el cuidado de los pies^(4,20,22–25,28, 30,31,33,37)^ (11;45,8 %), una mejora en las prácticas de autocuidado^(4,20,32,33,37)^ (5; 20,8 %) y cambios positivos en la actitud^(4,21)^ (2; 8,3 %).

Un estudio^(21)^ (4,1 %) investigó la eficacia de un video para prevenir complicaciones en los pies de pacientes con DM2 al aumentar el apoyo familiar y reveló que los videos educativos sobre el examen de los pies de pacientes con diabetes pueden modificar las actitudes y aumentar el apoyo familiar en el cuidado de los pies de estos pacientes.

En cuanto a los estudios que evaluaron videos en línea, los resultados describen la proporción de videos considerados útiles, moderadamente útiles o engañosos, así como las características de las fuentes productoras del contenido^(26,34–36)^(4; 16,6 %).

La Cuadro 3 presenta un resumen de los principales contenidos abordados, la intervención y los principales resultados de los estudios incluidos en la revisión.

**Cuadro 3:** Principales contenidos abordados, intervención y resultados de los estudios – Fortaleza, CE, Brasil, 2025.

ID	Nome	Tempo	Intervenção	Principais resultados
E1^(4)^	Verdadeiro ou Falso? e Diabetes e Cuidados diários com os pés	No consta	Un investigador visitó a los participantes en sus hogares. Durante las entrevistas, los participantes se encontraban acompañados por un familiar o conocido. Las preguntas se diseñaron para comprender la percepción de los usuarios sobre el cuidado de los pies antes y después de ver los videos.	Se identificaron tres categorías y dos subcategorías temáticas: 1) conocimiento previo, con las subcategorías fragilidad en las orientaciones profesionales y los videos como herramientas para recordar; 2) asimilación del lenguaje y el formato audiovisual; 3) posibilidades de impacto en el autocuidado
E2^(29)^	No consta	~6’	Se comparó un video instructivo (GI 1) con un folleto informativo sobre el cuidado de los pies con lectura guiada en tiempo real (GI 2) y la atención estándar (GC), en cuanto al cumplimiento y el conocimiento sobre el cuidado de los pies en personas con diabetes, así como en la percepción de los pacientes sobre la salud de sus pies a lo largo del tiempo.	No consta.
E3^(20)^	No consta	No consta	Se aplicaron pruebas previas y posteriores. En el GC, no se mostró ningún video educativo y se aplicó un cuestionario sobre el cuidado de los pies en las semanas 1 y 4. En el GI, por su parte, el cuestionario se aplicó a partir de la semana 1, y en las semanas 2 y 3 se mostró un video educativo sobre el cuidado de los pies. En la semana 4, se volvió a aplicar el cuestionario.	Los resultados de la prueba de Mann-Whitney antes de la proyección del video fueron de 0,957, sin que se observara una diferencia significativa entre el GI y el GC. Sin embargo, después de la proyección, el resultado fue de 0,000, lo que indica una diferencia significativa entre el GI y el GC. El uso de videos educativos en la educación aumentó significativamente el conocimiento y el autocuidado de los pies en pacientes con DM2.
E4^(15)^	“Pés de quem cuido”	17’	No se llevó a cabo	En el análisis de los IVC de todas las subcategorías, estos se consideraron válidos y confiables. La tercera versión del guion obtuvo un IVC de 0,94. Entre las principales consideraciones de los jueces durante el proceso de validación, se destaca la adecuación del lenguaje de los personajes y la reducción de los textos (diálogos de los personajes).
E5^(30)^	No consta	3’31”	Se aplicó un cuestionario sobre los conocimientos teóricos y prácticos relacionados con la prevención de lesiones en los pies en personas con DM. A continuación, los pacientes vieron el video. Ese mismo día se realizó una reevaluación de los conocimientos utilizando el mismo cuestionario.	Más del 90 % de las personas declararon estar «satisfechas» o «muy satisfechas» con el video educativo. Se observó una mejora significativa en el conocimiento sobre la prevención de lesiones en los pies en personas con DM. Aún queda por determinar el impacto del video en la reducción de la frecuencia de úlceras crónicas y amputaciones.
E6^(16)^	Autocuidado com os pés de idosos com diabetes	6’5”	No se realizó	En primer lugar, se analizaron 16 artículos que presentaban evidencia sobre la prevención de complicaciones en los pies de personas mayores con diabetes; posteriormente, se utilizó esta información como marco para la producción del video educativo. Tras la evaluación de nueve especialistas, se obtuvo un IVC general de 0,90.
E7^(34)^	No se aplica	Média de 6,8 ± 6,6’	No aplica	La categoría de contenido más frecuente fue el examen de los pies (58 videos), seguida de información específica sobre la enfermedad (53 videos). La fuente de subida más común fueron los médicos (28 %) y la menos común, los pacientes (1 %).
E8^(36)^	No consta	Média de 8,08 ± 13,27’	No aplica	De los videos evaluados, el 8 % se clasificó como muy útil, el 33,3 % como moderadamente útil, el 51,7 % como algo útil y el 6,9 % como no útil. Cabe destacar que el 4,6 % contenía información engañosa. El instructor era enfermero en solo el 12,6 % de estos videos.
E9^(17)^		24’7”	No se realizó	El guión del video presentó un IVC total de 0,95, con una confiabilidad interna de 0,849 según el alfa de Cronbach; los expertos en comunicación lo validaron con un IVC de 0,97 y el público objetivo con 0,95 (claridad) y 0,97 (relevancia).
E10^(18)^	“Ô Pé arretado”	2’51”	No se realizó	En la validación con jueces técnicos se observó un 100 % de concordancia en cuanto a la simplicidad, claridad y relevancia del contenido, y un CVCt de 1,0 para el guión del video de cordel. Además, hubo un 100 % de concordancia por parte de los evaluadores de la práctica clínica en cuanto a la simplicidad, claridad y relevancia del contenido y la apariencia del video «cordel», con concordancia en la adecuación del material educativo para el autocuidado en la prevención del pie diabético, así como de las complicaciones de la enfermedad.
E11^(19)^	“No rastro da prevenção: Uma narrativa sobre o diabetes e os cuidados com os pés na pessoa idosa”	4’33”	No se realizó	Se observó una concordancia unánime entre los especialistas para la mayoría de los criterios. No obstante, según el criterio adoptado para la validación del contenido del video —el IVC global, calculado a partir del promedio de los IVC de cada ítem—, no se consideró necesario realizar ninguna modificación en el contenido del video propuesto, ya que este se consideró válido con un 99,38 % de concordancia entre los evaluadores (IVC = 0,99).
E12^(21)^	No consta	12’	Se aplicó una prueba previa a través de WhatsApp y, a continuación, se envió el video educativo. Después de ver el video, los participantes realizaron el examen de los pies de manera independiente y enviaron un video con la grabación del examen que habían realizado. La prueba posterior se llevó a cabo en línea una semana después de la prueba previa, utilizando un formulario de Google.	Los videos educativos sobre el examen de los pies de pacientes con diabetes pueden modificar las actitudes y aumentar el apoyo familiar en el cuidado de los pies de estos pacientes. Con base en los resultados del análisis estadístico de Wilcoxon, el valor de p fue de 0,009 (p < 0,05), con una puntuación mediana de apoyo familiar total de 77 antes y de 81,6 después de la intervención.
E13^(22)^	No consta	5’15”	Se aplicaron una prueba previa y una prueba posterior en un solo grupo antes y después de ver el video del estudio de Abrar et al. (2020).	Los resultados revelaron que hubo un aumento en el nivel de conocimiento bueno (del 36,7 % al 90 %) y una disminución en el nivel de conocimiento moderado (del 56,7 % al 10 %) y en el nivel de conocimiento insuficiente (del 6,7 % al 0 %).
E14^(37)^	*Mobile Diabetic Foot Care Education* (M-DFCE)	6 seções, cada uma com duração aproximada de 1’30’’	GI: recibió orientación sobre el cuidado de los pies de los pacientes diabéticos a través de una aplicación móvil. GC: recibió orientación sobre el cuidado de los pies de los pacientes diabéticos de acuerdo con las directrices clínicas del hospital. El video se presentó una vez al inicio de la sesión de educación en salud y se realizó un seguimiento un mes después.	Hubo un aumento significativo en el nivel de conocimiento de los pacientes del GI sobre el cuidado de los pies (p = 0,001). No hubo un aumento significativo en la puntuación de conocimiento del GC entre la primera y la última evaluación (p = 0,905). No hubo diferencias entre los grupos en la evaluación inicial; sin embargo, los participantes del GI que recibieron M-DFCE con apoyo de animación presentaron niveles significativamente más altos de conocimiento, autoeficacia y comportamiento de cuidado de los pies que el GC.
E15^(23)^	Sin información	No consta	Aplicación de un video educativo sobre el cuidado de los pies (prueba previa y posterior)	El conocimiento de la muestra sobre el cuidado de los pies antes del video educativo tuvo un promedio de 6,40 ± 1,789, una mediana de 7,0, y la puntuación de conocimiento después del video alcanzó un promedio de 10,70 ± 0,571, con una mediana de 11,00 (p = 0,000).
E16^(24)^	No consta	>6’	La prueba previa se distribuyó a los participantes antes de la proyección del video. El video se reprodujo con un proyector durante menos de 6’. La prueba posterior se realizó con los mismos cuestionarios.	Los conocimientos de los participantes sobre el cuidado de los pies aumentaron significativamente después de ver los videos educativos en ambos idiomas, el makasarés (p = 0,001) y el bugis (p = 0,001).
E17^(26)^	No consta	>10’	No aplica	De los 87 videos incluidos, 56 (64,4 %) se clasificaron como útiles y 31 (35,6 %) como no útiles o engañosos. Se observó una diferencia significativa en la duración promedio de los videos útiles en comparación con los videos no útiles/engañosos (3,33 frente a 1,73 min; p < 0,0001). No hubo diferencias significativas en cuanto a las métricas de popularidad (me gusta, visualizaciones, suscripciones, etc.) entre los videos útiles y los no útiles/engañosos.
E18^(31)^	Sin información	9’	El GI recibió, como parte del módulo educativo sobre el cuidado de los pies, un video audiovisual. El GC recibió atención de rutina.	Las puntuaciones de conocimiento en el GI en la primera, segunda y tercera consultas fueron de 9,8 ± 1,8, 10,2 ± 1,6 y 11,0 ± 1,7, respectivamente. Las puntuaciones de conocimiento en el GC en la primera, segunda y tercera consultas fueron de 9,9 ± 1,7, 9,8 ± 1,6 y 10,0 ± 1,8, respectivamente. El cambio en la puntuación de conocimiento fue estadísticamente significativo (p < 0,001) en la tercera consulta en comparación con la primera en el grupo de intervención, pero no en el GC (p = 0,62). La puntuación de práctica también mejoró significativamente (p < 0,001) en el GI en la segunda consulta, pero no en el GC.
E19^(32)^	No consta	Sin información	La intervención consistió en una discusión interactiva en grupo utilizando diferentes materiales: un video narrativo, una presentación en PowerPoint y una guía impresa. Se realizaron pruebas previas y posteriores.	La puntuación promedio de cuidados de los pies aumentó de 3,5 ± 2,9 días a 5,9 ± 1,8 días un mes después de la intervención (variación promedio de 2,4 ± 3,1 días; p < 0,001). Se observó una variación favorable en 75 (37,7 %) participantes.
E20^(35)^	No consta	Hasta 30’	No aplica	Las dos prácticas de cuidado de los pies más mencionadas en los videos fueron «revisar los pies diariamente» (n = 34; 38,20 %) y «usar zapatos y calcetines adecuados» (n = 26; 29,21 %). Solo el 14,6 % de los videos contenía información útil, sin contar la información parcialmente útil. Ese 14,6 % provenía de podólogos o clínicas especializadas en pies. Solo el 10,1 % del total de videos correspondía a usuarios en general.
E21^(25)^	“Guidance Motion Picture” (GMP)	No consta	El GI (orientación sobre la película + folleto informativo), el GC positivo 1 (orientación sobre la película) y el GC positivo 2 (folleto informativo).	El conocimiento fue significativo para el GI (p = 0,005), para el GC positivo 1 (p = 0,011) y para el GC positivo 2 (p = 0,097). El análisis de la actitud fue significativo para todos los grupos (p = 0,005). El análisis de la acción fue significativo para el GI (p = 0,005), el GC positivo 1 (p = 0,005) y el GC positivo 2 (p = 0,011). Existen diferencias en la eficacia entre la combinación de video educativo + folleto informativo y el video educativo por sí solo, en relación con el aumento del conocimiento (p = 0,047), la actitud (p = 0,022) y la acción (p = 0,037).
E22^(27)^	No consta	~10’	Los participantes se dividieron al azar en dos grupos del mismo tamaño: el G1 vio la versión del video educativo centrada en las ganancias. El G2 vio la versión centrada en las pérdidas. Cada participante completó cuatro sesiones de evaluación: antes de ver el video (datos demográficos, actitud, conocimiento y comportamiento), inmediatamente después de ver el video (actitud y conocimiento) y en seguimientos a los 3 y 6 meses (actitud, conocimiento y comportamiento).	Los videos con mensajes de prevención centrados en las ganancias son más eficaces que los mensajes centrados en las pérdidas para promover comportamientos que mantienen la salud en personas con diabetes.
E23^(28)^	No consta	No consta	Los participantes se dividieron en tres grupos. El GC recibió el material educativo escrito que actualmente proporciona el hospital a los pacientes con diabetes. El GI recibió educación en video que abarcaba el mismo contenido que el material escrito. Un tercer grupo recibió educación tanto por escrito como en video. El video se vio de 1 a 3 veces mientras la persona se encontraba en el hospital	La mayoría de los pacientes presentó un cambio significativo en las pruebas posteriores a la educación al utilizar la herramienta de educación en video (GI).
E24^(33)^	No consta	28’	Para evaluar la utilidad del programa, se elaboró un cuestionario. Se pidió a los pacientes que respondieran las mismas ocho preguntas (sí/no) antes y después de ver el video.	Después de ver el video, la cantidad de personas que supieron responder las preguntas sobre el cuidado de los pies aumentó del 69 % al 91 %. Al cabo de seis meses, la mayoría destacó que había seguido al menos algunas de las recomendaciones de autocuidado de los pies que no habían puesto en práctica anteriormente.

Abreviaturas: DM: diabetes mellitus; DM2: diabetes mellitus tipo 2; IVC: Índice de validez de contenido; CVC: Coeficiente de validez de contenido; CVCt: Coeficiente de validez de contenido total; GC: grupo de control; GI: grupo de intervención; DE: desviación estándar; GMP: Guidance Motion Picture; M-DFCE: Educación móvil sobre el cuidado del pie diabético.

Fuente: Autores (2025).

En cuanto al contenido abordado en los videos, los estudios presentaron orientaciones generales y sobre la diabetes^(4,15,16,18,19,25,26,30,31,34–36)^ (12; 50,0 %), el autocuidado de los pies, en general, sin especificar las orientaciones^(4,16–18,20,23,25–27,29–31,37)^ (13; 54,1 %). En cuanto a las acciones de autocuidado descritas en el video, predominó el cuidado de las uñas^(15–17,19,22,24,26,30,32,35,36)^ (11; 45,8 %), seguidas de la inspección de los pies^(15–17,22,24,26,30,34–36)^, el lavado de los pies^(15–17,22,24,26,30,32,35,36)^, el uso adecuado de calzado^(15–17,19,22,24,26,30,35,36)^y el uso de medias^(15–17,22,24,26,30,32,35,36)^ (10; 41,6 % cada uno). El contenido menos abordado en los videos fue la orientación sobre ejercicios para los pies^(16,26)^ (2; 8,3 %). Tres estudios^(21,28,33)^ (12,5 %) no mencionaron el contenido del video.

La Figura 2 presenta un resumen de los contenidos abordados en los videos sobre el autocuidado de los pies en los estudios de la revisión de alcance.

**Figura 2:** Resumen de los contenidos abordados en los videos sobre el autocuidado de los pies en los estudios de la revisión de alcance – Fortaleza, CE, Brasil, 2025.

**Fuente:** Autores (2025).

## DISCUSIÓN

Los hallazgos de esta revisión de alcance evidencian el uso creciente de videos educativos como estrategia de apoyo al autocuidado de las personas con diabetes, especialmente en el cuidado de los pies, un aspecto esencial para la prevención de úlceras y amputaciones^(38,39)^. Estos recursos tienen el potencial de ampliar el acceso a la información en salud, fomentar el protagonismo del paciente y fortalecer las acciones educativas en diferentes contextos de atención.

A pesar de este potencial, los estudios incluidos identificaron debilidades relacionadas con la calidad de la información disponible. Se observó una elevada heterogeneidad, la ausencia de validación clínica y de directrices estandarizadas en cuanto al contenido, la duración, el lenguaje, las estrategias pedagógicas y los recursos audiovisuales utilizados, además de diferencias en los criterios empleados para la evaluación de los materiales. Los estudios que analizaron videos disponibles en plataformas abiertas, como YouTube, demostraron una mayor variabilidad en la calidad científica y una menor alineación con las recomendaciones basadas en la evidencia, en comparación con los materiales producidos por instituciones de salud o grupos académicos^(40,41)^.

Además, muchos videos presentaban información incompleta o superficial sobre medidas preventivas fundamentales para el autocuidado de los pies, incluyendo la inspección diaria, la higiene adecuada, la evaluación del calzado y la identificación temprana de lesiones. Se observó que la mayoría de los materiales no contemplaban íntegramente las recomendaciones del *International Working Group on the Diabetic Foot* ^(1)^, lo que limitaba el potencial educativo y la aplicabilidad clínica de estas tecnologías.

Los estudios también pusieron de manifiesto diferencias importantes en cuanto a los procesos de desarrollo y validación de los videos. Los materiales sometidos a la evaluación de especialistas y a pruebas con usuarios presentan mayor claridad, organización y adecuación de la información, lo que favorece la comprensión y la aplicabilidad de las orientaciones educativas^(30,42,43)^. Además, se observó que gran parte de los videos disponibles en plataformas digitales no pasa por ningún proceso de validación, lo que refuerza la urgencia de incorporar etapas de evaluación clínica como fase obligatoria en el desarrollo de materiales educativos.

En este contexto, se destaca la necesidad de protocolos estandarizados para la producción de videos educativos en salud. Aunque existen recomendaciones generales sobre educación en salud y comunicación digital, los estudios señalan una escasez de modelos sistematizados que orienten aspectos como la elaboración de guiones, la definición del lenguaje, el uso de recursos audiovisuales y la adaptación del contenido a las necesidades del público objetivo^(44,45)^. Esta falta de estandarización dificulta la comparación entre estudios, compromete la reproducibilidad de las intervenciones y limita la consolidación de la evidencia sobre la efectividad de los videos educativos.

Otro aspecto relevante se refiere al impacto de la desigualdad en el acceso a las tecnologías educativas. Aunque los videos pueden ampliar el alcance de las acciones de educación en salud, factores como un bajo nivel socioeconómico, el acceso limitado a Internet y las dificultades de alfabetización digital pueden restringir su uso por parte de las poblaciones más vulnerables^(46,47)^. Por lo tanto, la incorporación de tecnologías digitales en la educación en salud debe ir acompañada de políticas públicas orientadas a la inclusión y a la ampliación del acceso a las herramientas tecnológicas.

En el contexto brasileño, se observa un avance en la incorporación de la Salud Digital como eje estructurante del Sistema Único de Salud (SUS), especialmente a través de iniciativas como la Política Nacional de Salud Digital y el Conecte SUS, que estimulan el desarrollo y el uso de recursos tecnológicos aplicados a la atención en salud^(48,49)^. Sin embargo, los hallazgos de esta revisión demuestran que la expansión de estas herramientas debe ir de la mano con el fortalecimiento de los mecanismos de evaluación de la calidad de la información proporcionada y detallada.

En este sentido, un estudio de Smith et al.^(26)^, al evaluar videos sobre el autocuidado de los pies en personas con diabetes disponibles en YouTube, identificó que los videos más largos fueron calificados significativamente como útiles en comparación con los considerados engañosos o poco útiles. Este resultado sugiere que los materiales más detallados pueden favorecer un mayor alcance de las orientaciones educativas, aunque la duración, por sí sola, no sea suficiente para garantizar la calidad científica.

Además de la calidad técnica, los estudios refuerzan la importancia de fortalecer la capacidad crítica de los usuarios frente a la información de salud difundida en el entorno tecnológico. La amplia circulación de contenido sin revisión científica puede dificultar la identificación de fuentes confiables, lo que hace esencial el desarrollo de estrategias educativas que promuevan la alfabetización digital y el uso crítico de las tecnologías en salud^(20,47,49)^.

A pesar de las limitaciones identificadas, los videos educativos siguen siendo herramientas prometedoras para apoyar el autocuidado de los pies en personas con diabetes, especialmente cuando se utilizan como complemento de las orientaciones presenciales brindadas por los profesionales de la salud. Su impacto positivo depende directamente de la calidad del contenido, la validación clínica, el uso de estrategias pedagógicas adecuadas y la ampliación del acceso a las tecnologías.

Los hallazgos evidencian la necesidad de desarrollar videos educativos de orientación para el autocuidado de los pies en consonancia con las directrices actuales, adaptados culturalmente y capaces de atender las diferentes necesidades y contextos socioculturales de las personas con diabetes. Además, resaltan la importancia de ampliar el acceso a las tecnologías digitales como estrategia para potenciar las acciones de educación en salud, fomentar el compromiso con el autocuidado y fortalecer la autonomía de las personas con diabetes en la prevención de complicaciones relacionadas con los pies.

Las principales limitaciones de esta revisión de alcance incluyen la heterogeneidad metodológica de los estudios incluidos, lo que dificultó la comparación directa entre los resultados, especialmente en lo que se refiere a los diferentes formatos, duraciones y criterios de evaluación de los videos educativos. Además, muchos de los materiales analizados no presentaban información suficiente sobre los procesos de validación o la base teórica utilizada, lo que limitó la evaluación de la calidad y la credibilidad de los contenidos.

El predominio de estudios publicados en determinados idiomas puede haber llevado a una subrepresentación de producciones procedentes de países de ingresos bajos y medios, donde la desigualdad digital es más marcada.

Por último, es posible que la búsqueda en bases de datos no haya captado videos difundidos exclusivamente en plataformas digitales no indexadas, lo que podría haber dado lugar a la pérdida de materiales relevantes; esto sugiere que los hallazgos deben interpretarse con cautela.

## CONCLUSIÓN

Los videos educativos disponibles y descritos en la literatura representan un recurso potencialmente valioso para fortalecer el autocuidado de los pies en personas con diabetes. Tienen el potencial de fortalecer el autocuidado y ampliar el acceso a la educación en salud. Sin embargo, su efectividad depende de una mayor estandarización, una selección profesional y procesos de validación.

Invertir en materiales audiovisuales basados en evidencia y alineados con las guías clínicas es fundamental para mejorar la calidad, la seguridad y la aplicabilidad de estas tecnologías en la práctica clínica y en la atención a las personas con diabetes.

## DISPONIBILIDAD DE DATOS

Todo el conjunto de datos que respalda los resultados de este estudio se publicó en el propio artículo.
